# Ganoderic acid D prevents oxidative stress‐induced senescence by targeting 14‐3‐3ε to activate CaM/CaMKII/NRF2 signaling pathway in mesenchymal stem cells

**DOI:** 10.1111/acel.13686

**Published:** 2022-08-05

**Authors:** Huan Yuan, Yan Xu, Yi Luo, Jia‐Rong Zhang, Xin‐Xin Zhu, Jian‐Hui Xiao

**Affiliations:** ^1^ Institute of Medicinal Biotechnology Affiliated Hospital of Zunyi Medical University Zunyi China; ^2^ Zunyi Municiptal Key Laboratory of Medicinal Biotechnology and Guizhou Provincial Research Center for Translational Medicine Affiliated Hospital of Zunyi Medical University Zunyi China

**Keywords:** 14‐3‐3ε, aging mouse model, anti‐aging, bone‐marrow mesenchymal stem cells, CaM/CaMKII/Nrf2 signaling, ganoderic acid D, human amniotic mesenchymal stem cells

## Abstract

Stem cell senescence is an important cause of aging. Delaying senescence may present a novel way to combat aging and age‐associated diseases. This study provided a mechanistic insight into the protective effect of ganoderic acid D (GA‐D) against human amniotic mesenchymal stem cell (hAMSCs) senescence. GA‐D, a *Ganoderma lucidum*‐derived triterpenoid, markedly prevented hAMSCs senescence via activating the Ca^2+^ calmodulin (CaM)/CaM‐dependent protein kinase II (CaMKII)/nuclear erythroid 2‐related factor 2 (Nrf2) axis, and 14‐3‐3ε was identified as a target of GA‐D. 14‐3‐3ε‐encoding gene (*YWHAE*) knockdown in hAMSCs reversed the activation of the CaM/CaMKII/Nrf2 signals to attenuate the GA‐D anti‐aging effect and increase senescence‐associated *β*‐galactosidase (SA‐*β*‐gal), p16 and p21 expression levels, including reactive oxygen species (ROS) production, thereby promoting cell cycle arrest and decreasing differentiation potential. *YWHAE* overexpression maintained or slightly enhanced the GA‐D anti‐aging effect. GA‐D prevented *d‐galactose‐caused* aging in mice by significantly increasing the total antioxidant capacity, as well as superoxide dismutase and glutathione peroxidase activity, and reducing the formation of malondialdehyde, advanced glycation end products, and receptor of advanced glycation end products. Consistent with the protective mechanism of GA‐D against hAMSCs senescence, GA‐D delayed the senescence of bone‐marrow mesenchymal stem cells in this aging model in vivo, reduced SA‐*β*‐gal and ROS production, alleviated cell cycle arrest, and enhanced cell viability and differentiation via regulating 14‐3‐3ε and CaM/CaMKII/Nrf2 axis. Therefore, GA‐D retards hAMSCs senescence by targeting 14‐3‐3ε to activate the CaM/CaMKII/Nrf2 signaling pathway. Furthermore, the in vivo GA‐D anti‐aging effect may involve the regulation of stem cell senescence via the same signal axis.

## Graphical abstract

GA‐D prevents MSC senescence via regulating 14‐3‐3e to activate the CaM/CaMKII/Nrf2 pathway; GA‐D prevents d‐gal‐caused aging in mice by enhancing antioxidative defense and retards the BMSCs senescence in d‐gal‐caused aging mice; GA‐D may be a potential anti‐aging agent.

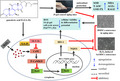

**Abbreviations**
AGE: advanced glycation end product
BMSC: bone‐marrow mesenchymal stem cell
BSA: bovine serum albumin
CaM: calmodulin
CaMKII: CaM‐dependent protein kinase II
CaMKK2: CaMK kinase 2
CCK8: Cell Counting Kit‐8
CETSA: cellular thermal shift assay
DARTS: drug affinity‐responsive target stability
d‐gal: d‐galactose
DMSO: dimethyl sulfoxide
D‐PBS: Dulbecco's phosphate‐buffered saline
DS: developmental senescence
EIF5A: eukaryotic translation initiation factor 5A
FBS: fetal bovine serum
GA‐D: ganoderic acid D
GSH‐Px: glutathione peroxidase
H_2_O_2_
: hydrogen peroxide
hAMSC: human amniotic mesenchymal stem cell
HSC: hematopoietic stem cell
LG‐DMEM: low‐glucose Dulbecco's modified Eagle's medium
MDA: malondialdehyde
MG: model group
MOI: multiplicity of infection
NG: normal group
Nrf2: nuclear erythroid 2‐related factor 2
OIS: oncogene‐induced senescence
PFA: paraformaldehyde
PPI: protein–protein interactions
RAGE: receptor for advanced glycation end product
ROS: reactive oxygen species
RS: replicative senescence
SA‐*β*‐gal: senescence‐associated *β*‐galactosidase
SDS‐PAGE: sodium dodecyl sulfate‐polyacrylamide gel electrophoresis
SIPS: stress‐induced premature senescence
SOD: superoxide dismutase
T‐AOC: total antioxidant capacity
WB: Western blotting

## 1 INTRODUCTION

Aging, a phenomenon of physiological dysfunction in an organism, has been regarded as the primary reason for chronic and degenerative diseases such as cancer, diabetes, atherosclerosis, and Alzheimer's disease (Wu et al., 2021). Exploration of the basic factors of organismal senility to prevent and treat age‐related diseases has become increasingly important in recent years. Adult stem cells in organisms, as the source of seed cells for the repair of injured tissues and organs, are the major cells that maintain tissue homeostasis and regeneration (Boyette & Tuan, 2014; Zhang et al., 2016). However, senescent stem cells lose the ability to repair damaged tissues, impair the homeostasis in the tissue, and even cause age‐related diseases. In the organisms, increasing evidence indicates that aging may cause the senescence and reduce the number of stem cells (Cianflone et al., 2020; Díaz‐Morenoa et al., 2018; Tian & Li, 2014; Vilas et al., 2018; Vyver et al., 2021; Yi et al., 2021). For instance, hematopoietic stem cells (HSCs) are incapable of regenerating the damaged hematopoietic system as efficiently as young HSCs, which eventually leads to hematopoietic dysfunction and other related diseases (Chen & Ju, 2019). In addition, a reduction in adult hippocampal neurogenesis has been detected in age‐associated degenerative diseases, in which one of the key mechanisms underlying this reduction in neurogenesis is stem cell senescence (Bedrosian et al., 2021). Therefore, stem cell senescence and depletion are important signs of aging, and we hypothesize that the model of stem cell senescence may be used as a new approach for discovering anti‐aging agents.

*Ganoderma lucidum*, a prominent Chinese traditional medicine, has been used as an anti‐aging elixir in China and other oriental countries for thousands of years. In addition, many studies have revealed the therapeutic potential of *G. lucidum*, and more than 400 distinct chemical entities, including polysaccharides, triterpenoids, and sterols, have been isolated from *G. lucidum*, especially triterpenoids that comprise more than 150 different chemical structures (Liang et al., 2019; Zhong & Xiao, 2009). *G. lucidum* is a triterpenoid bioresource. However, only ethanol and water extracts of *G. lucidum*, isolated polysaccharides, and several ergosterol derivatives have lifespan elongation or anti‐aging effects (Wang et al., 2017). These studies revealed that prolonging lifespan is just the tip of the iceberg because the number of chemical entities with anti‐aging activities that have been reported is less than the number of known *G. lucidum*‐derived compounds. Moreover, little is known regarding the anti‐aging effects of *G. lucidum* triterpenoids. *G. lucidum* triterpenoids exhibit potent antioxidant properties and free radical scavenging activities, indicating that they might exert potential lifespan extension effects. Recently, we found that ganoderic acid D (GA‐D), a triterpenoid compound produced by *G. lucidum*, obviously alleviates the senescence of stem cells via the PERK/nuclear erythroid 2‐related factor 2 (Nrf2) signals (Xu et al., 2020). However, the upstream regulators and downstream effectors of the Nrf2 pathway and possible targets of GA‐D in protecting human amniotic mesenchymal stem cells (hAMSCs) from senescence are still largely elusive and its anti‐aging effect in vivo remains unclear.

As previously described, GA‐D could inhibit cancer cell proliferation via the mTOR signaling pathway, inducing cell cycle arrest, apoptosis, and autophagy, and regulate the reprogramming of energy metabolism through SIRT3/cyclophilin D (Liu et al., 2018; Shao et al., 2020; Yue et al., 2010). Moreover, the possible targets of GA‐D to exert its cytotoxic effect against cancer cells may involve six isoforms of the 14‐3‐3 family (14‐3‐3β/α, 14‐3‐3γ, 14‐3‐3ζ/δ, 14‐3‐3ε, 14‐3‐3θ, and 14‐3‐3σ), annexin A5, aminopeptidase B, eukaryotic translation initiation factor 5A, and peroxiredoxin 2 (Yue et al., 2008, 2010). The above six isoforms of the 14‐3‐3 family can bind directly to GA‐D, as predicted by the INVDOCK program (Yue et al., 2008). Although INVDOCK is widely employed to predict the molecular targets and mechanisms of different functional compounds, their binding energies cannot be normalized to appropriately sort candidate targets (Kharkar et al., 2014). Thus, the targets predicted by INVDOCK require further confirmation. There is currently insufficient evidence to confirm that these 14‐3‐3 targets are associated with the anticancer activity of GA‐D. The highly conserved 14‐3‐3 proteins originate from a dimeric phosphoserine‐binding eukaryotic protein family and consist of seven members (β/α, γ, δ/ζ, ε, η, θ, and σ) in mammals. Moreover, these proteins can bind to hundreds of protein targets in a phosphoserine‐dependent or phosphoserine‐independent manner to assist protein folding, stimulation, and/or inhibition of other protein–protein interactions (PPI), thereby regulating many vital molecular and cellular events, such as protein trafficking, signal transduction, mitogenesis, cell cycle, survival, and apoptosis (Morrison, 2009; Stevers et al., 2018). Furthermore, they exhibit similar expression paradigms in cancer, aging, and degenerative disorders; thereby, they are valuable drug targets, indicating great therapeutic potential in targeting 14‐3‐3 proteins (Aghazadeh & Papadopoulos, 2016; Fan et al., 2019). Therefore, these previous reports suggest that 14‐3‐3 proteins may be suitable targets of GA‐D in protecting hAMSCs from senescence.

In the present study, we further explored the target and mechanism of GA‐D in protecting hAMSCs from senescence according to our previous study (Xu et al., 2020) and explored the anti‐aging effect at the molecular and cellular levels, as well as in a d‐galactose (d‐gal)‐caused aging murine model. These studies provide not only a scientific basis for the development of GA‐D as an anti‐aging agent but also a new paradigm for the discovery of anti‐aging agents by the stem cell senescence model. Our results provide scientific evidence for the theory of stem cell aging, indicating that anti‐aging agents may target the senescence of adult stem cells in organisms.

## 2 RESULTS

### 2.1 GA‐D prevents hAMSCs senescence by targeting 14‐3‐3ε

**FIGURE 1:**
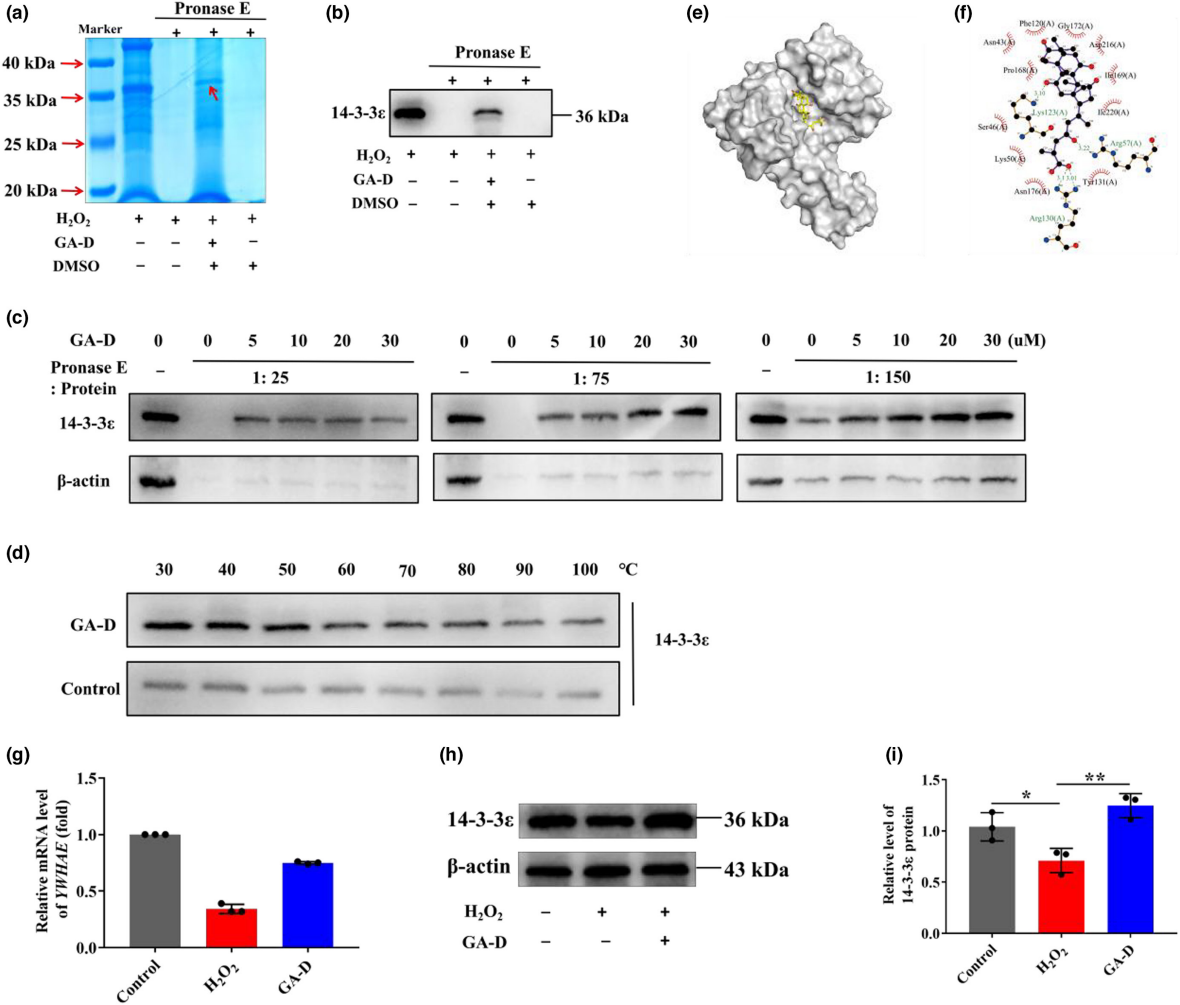
GA‐D prevents H_2_O_2_‐induced premature hAMSCs senescence through targeting 14‐3‐3ε. (a) Coomassie (SimplyBlue) staining after DARTS assay. The red arrow indicates the undigested protein bound to GA‐D. (b) Protein bands after DARTS assay, showing the binding of small‐molecule GA‐D to the target protein 14‐3‐3ε. (c) Target validation by DARTS assay under different conditions. (d) Target validation by CETSA assay. (e) Theoretical binding model of GA‐D to the 14‐3‐3ε‐binding pocket. The ball and stick model of GA‐D; the protein is displayed in gray. (f) Specific interaction modes between GA‐D and 14‐3‐3ε. (g) Changes in 14‐3‐3ε‐encoding gene (*YWHAE*) mRNA expression in H_2_O_2_‐treated hAMSCs after GA‐D pretreatment. (h) Changes in 14‐3‐3ε expression in H_2_O_2_‐treated hAMSCs after GA‐D treatments. (i) The relative expression level of 14‐3‐3ε in H_2_O_2_‐treated hAMSCs after GA‐D treatments. N = 3. Control, control group; H_2_O_2_, senescent group; GA‐D, GA‐D treatment group; mock‐vehicle, GA‐D treatment group plus empty carrier; h‐14‐3‐3ε, GA‐D treatment group plus *YWHAE* overexpression; sh‐14‐3‐3ε, GA‐D treatment group plus *YWHAE* knockdown; $\bar{x}\pm \mathrm{SD}$, mean ± standard deviation; protease E, *Streptomyces* proteinase. *p < 0.05, **p < 0.01. CETSA, cellular thermal shift assay; DARTS, drug affinity‐responsive target stability; DMSO, dimethylsulfoxide; GA‐D, ganoderic acid D; H_2_O_2_, hydrogen peroxide; hAMSC, human amniotic mesenchymal stem cell; ROS, reactive oxygen species.

The hAMSCs highly expressed surface markers of MSCs, including CD29 (98.98%), CD73 (99.90%), CD90 (98.14%), and CD105 (82.53%), whereas it did not express those of HSCs, including CD34, CD11b, CD19, CD45, and HLA‐DR (Figure S1a). In addition, vimentin, a marker protein of MSCs, was strongly expressed in hAMSCs, but not an epithelial cell marker cytokeratin 19 (Figure S1b). The characteristics of hAMSCs meet the MSC certification standards recommended by the International Society for Cellular Therapy (Dominici et al., 2006). GA‐D exerted protective effects against hydrogen peroxide (H_2_O_2_)‐induced senescence in hAMSCs. The morphology of senescent hAMSCs changed from flat to normal fusiform after GA‐D treatment (Figure S2a). At the same time, the production of *β*‐galactosidase was suppressed under 10 μM GA‐D treatment (Figure S2b), directly causing a decrease in the proportion of the senescence‐associated *β*‐galactosidase (SA‐*β*‐gal)‐positive cells, from 41.26 ± 3.16% to 15.55 ± 1.30% (Figure S2c). In addition, the expression of p16 and p21 proteins was markedly decreased in the H_2_O_2_‐treated hAMSCs after GA‐D treatment (Figure S2d–f). Here, GA‐D treatment had no impact on the surface markers of H_2_O_2_‐treated hAMSCs (Figure S4). The dimethyl sulfoxide (DMSO) group showed no changes in hAMSCs senescence (Figure S2a–c). Subsequently, the transcriptional levels of the possible target proteins of GA‐D, including five 14‐3‐3 proteins (14‐3‐3β/α, 14‐3‐3θ, 14‐3‐3σ, 14‐3‐3γ, and 14‐3‐3ε), annexin A5, and eukaryotic translation initiation factor 5A (EIF5A) were analyzed in hAMSCs (Yue et al., 2008, 2010). Interestingly, only the expression level of *YWHAE* (14‐3‐3ε‐encoding gene) was significantly altered in the presence of GA‐D (Figure S3a). Subsequently, the interaction between GA‐D and 14‐3‐3ε isoform in hAMSCs was validated using drug affinity‐responsive target stability (DARTS) and Western blotting (WB) assays. In the absence of protease E, all protein bands were detected with different intensities in the H_2_O_2_ group, but these bands were no longer present after the addition of pronase E (Figure 1a). However, a protein band was detected at 36 kDa after adding GA‐D instead of DMSO (Figure 1a), and the WB assay showed that 14‐3‐3ε isoform was present (Figure 1b), implying that GA‐D might be combined with 14‐3‐3ε isoform and prevent 14‐3‐3ε isoform from being hydrolysis. To validate the 14‐3‐3ε as the direct interacting protein target of GA‐D, two typical target identification approaches, including DARTS by pronase‐mediated proteolytic digestion and cellular thermal shift assay (CETSA) by thermal stability shift, were employed. These approaches mainly use WB as a readout. In the presence of GA‐D at concentrations of 5, 10, 20, and 30 μM, similar‐sized protected bands are shown in Figure 1c. Protected bands were not observed in the absence of GA‐D under the proteolytic digestion with moderate to high pronase concentrations. With the increase of GA‐D concentration, DARTS detection revealed strong protected bands in a dose‐dependent manner under the proteolytic digestion with low to moderate concentrations of pronase (pronase:protein ratios were 1:150 and 1:75; Figure 1c). Therefore, these data indicate that GA‐D can bind 14‐3‐3ε protein to protect 14‐3‐3ε from digestion. In CETSA, furthermore, when the temperature was within the range of 30–80°C, the expression level of the 14‐3‐3ε isoform in the GA‐D treatment group was significantly higher than that in the control group, indicating that GA‐D enhanced the thermal stability of 14‐3‐3ε isoform (Figure 1d). Taken together, these results suggest that GA‐D could enhance the enzymatic hydrolysis stability and thermal denaturation stability of 14‐3‐3ε isoform, indicating that there is a specific binding between GA‐D and 14‐3‐3ε isoform. Next, we further analyzed the binding sites between GA‐D and 14‐3‐3ε isoform using molecular docking Autodock software. The result indicated that GA‐D bound tightly to the 14‐3‐3ε capsule (Figure 1e). There was a hydrophobic interaction between GA‐D and residues Phe120, Gly172, Asp216, Ile220, Tyr131, Asn176, Lys50, Ser46, Pro168, and Asn43 of 14‐3‐3ε protein. Simultaneously, GA‐D formed four hydrogen bonds with 14‐3‐3ε residues Lys123, Arg57, and Arg130 to ensure the stable binding of GA‐D to the 14‐3‐3ε isoform (Figure 1f). These data suggest that the 14‐3‐3ε isoform might be a potential target of GA‐D for preventing the senescence of hAMSCs. In addition, 14‐3‐3ε expression at both mRNA and protein levels was significantly upregulated after GA‐D treatment (Figure 1g–i), indicating that GA‐D not only binds to 14‐3‐3ε but also regulates its expression.

### 2.2 Anti‐senescence effect of GA‐D is changed upon knockdown and overexpression of 14‐3‐3ε

**FIGURE 2:**
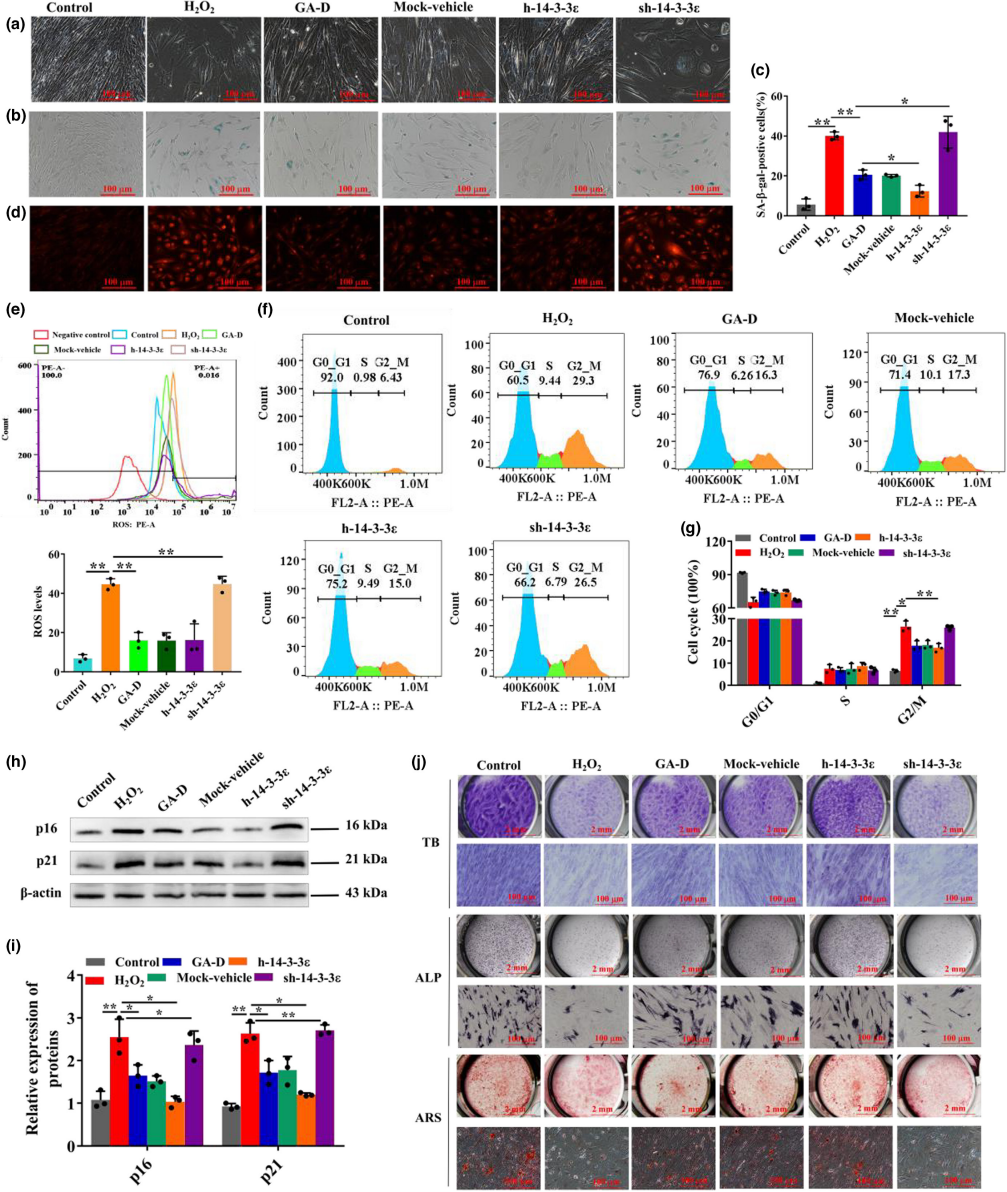
Effect of GA‐D on biological characteristics of senescent hAMSCs induced by H_2_O_2_ by upregulating *YWHAE* expression. (a) Changes in the morphology of hAMSCs. (b) The production of *β*‐galactosidase. (c) The number of SA‐β‐gal positive cells. (d) The production of intracellular ROS levels by confocal measurement. (e) The levels of intracellular ROS by FACS measurement. (f) Cell cycle distribution of hAMSCs through flow cytometry after different treatments. (g) The number of hAMSCs at different stages of the cell cycle. (h) The expression of p16 and p21 was detected by western blotting. (i) Relative expression levels of p16 and p21. (j) Chondrocyte differentiation by toluidine blue (TB) staining, osteoblast differentiation by alkaline phosphatase (ALP) and alizarin red S (ARS) stainings. Scale bar: 2 mm, 100 μm. N = 3. Control, control group; H_2_O_2_, senescent group; GA‐D, GA‐D treatment group; mock‐vehicle, GA‐D treatment group plus empty carrier; h‐14‐3‐3ε, GA‐D treatment group plus *YWHAE* overexpression; sh‐14‐3‐3ε, GA‐D treatment group plus *YWHAE* knockdown; $\bar{x}\pm \mathrm{SD}$, mean ± standard deviation. *p < 0.05, **p < 0.01. GA‐D, ganoderic acid D; H_2_O_2_, hydrogen peroxide; hAMSC, human amniotic mesenchymal stem cell; ROS, reactive oxygen species.

To verify whether the 14‐3‐3ε‐encoding gene (*YWHAE*) was involved in the anti‐senescent effect of GA‐D in H_2_O_2_‐induced senescent hAMSCs, *YWHAE* was knocked down and overexpressed via adenovirus transfection. The fluorescence intensity reached 80% when the multiplicity of infection (MOI) was 200 (Figure S3b). *YWHAE* expression was significantly upregulated in the h‐14‐3‐3ε group and downregulated in the sh‐14‐3‐3ε group after 24, 48, and 72 h (Figure S3c,d). After *YWHAE* knockdown and overexpression, no difference in the expression of hAMSCs surface markers was observed compared with GA‐D group (Figure S4). However, after the knockdown of *YWHAE*, the morphology of hAMSCs became flat compared with that in the GA‐D group but remained normal after *YWHAE* overexpression (Figure 2a). Meantime, the generation of *β*‐galactosidase and the proportion of the SA‐*β*‐gal‐positive cells in the sh‐14‐3‐3ε group increased significantly compared to the GA‐D group, but *YWHAE* overexpression decreased this effect (Figure 2b,c). Moreover, the formation of intracellular reactive oxygen species (ROS) was also obviously increased after *YWHAE* knockdown, while there was no change in intracellular ROS production in the h‐14‐3‐3ε group compared with that of the GA‐D group (Figure 2d,e). These data suggest that 14‐3‐3ε knockdown significantly inhibits the positive effect of GA‐D on senescent hAMSCs.

### 2.3 Effects of GA‐D on cell cycle arrest and differentiation of senescent hAMSCs after knockdown and overexpression of YWHAE

Cell cycle arrest and differentiation potential were determined to further confirm that 14‐3‐3ε mediates the anti‐senescent effect of GA‐D on hAMSCs. The cell cycle of senescent hAMSCs in the H_2_O_2_ group was arrested at the G2/M phase, increasing the cell proportions in the G2 phase from 6.43 ± 0.16% to 29.3 ± 0.28% (Figure 2f,g). Our results show that this change in the cell cycle was partially recovered under cell proportions of 16.3 ± 0.28% to 15.0 ± 0.20% in the GA‐D and h‐14‐3‐3ε groups, respectively. However, the cell cycle was hardly rescued in the sh‐14‐3‐3ε group (Figure 2f,g). In our results, GA‐D caused a significant decrease in the expression of cyclin‐dependent kinase inhibitors, including p16^INK4a^ and p21, in senescent hAMSCs. However, this effect of GA‐D weakened when *YWHAE* was knocked down or became stronger when it was overexpressed in senescent hAMSCs (Figure 2h,i). Furthermore, GA‐D significantly enhanced the differentiation potential of senescent hAMSCs compared with that of the H_2_O_2_ group, increasing the formation of cartilage marker glycosaminoglycan, early osteogenic marker alkaline phosphatase, and late osteogenic marker calcium nodule (Figure 2j), and lipid droplets (Figure S3e). However, GA‐D no longer improved the differentiation potential of senescent hAMSCs when *YWHAE* was knocked down (Figure 2j; Figure S3e). The h‐14‐3‐3ε group showed no change in comparison with the GA‐D group. These data indicate that the 14‐3‐3ε isoform is essential for GA‐D to prevent H_2_O_2_‐induced senescent hAMSCs.

### 2.4 YWHAE mediates the anti‐senescent effect of GA‐D on hAMSCs through CaM/CaMKII and Nrf2/HO‐1/NQO1 signaling pathways

**FIGURE 3:**
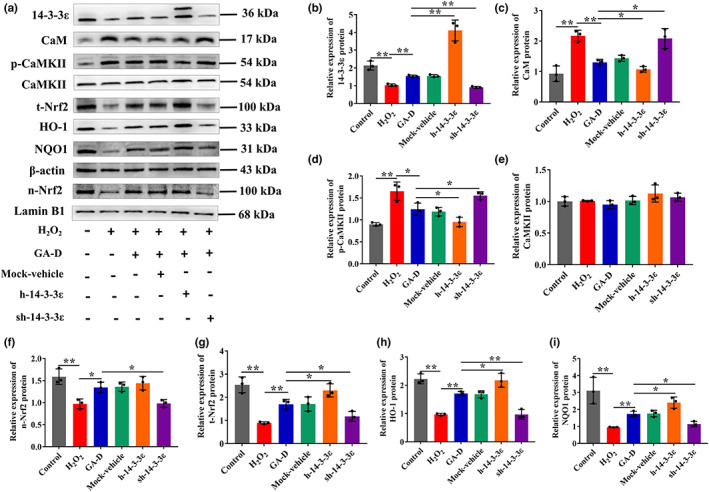
CaM/CaMKII and Nrf2/HO‐1/NQO1 signaling pathways were attenuated after *YWHAE* knockdown. (a) Changes in 14‐3‐3ε, t‐Nrf2, n‐Nrf2, HO‐1, NQO1, CaM, p‐CaMKII, CaMKII, p16^INK4a^, and p21 expression in hAMSCs upon different treatments. (b–i) Relative expression levels of 14‐3‐3ε, n‐Nrf2, t‐Nrf2, CaM, CaMKII, p‐CaMKII, HO‐1, NQO1, p16^INK4a^, and p21. N = 3. N‐Nrf2, nuclear Nrf2; t‐Nrf2, total Nrf2; p‐CaMKII, phosphorylated CaMKII; control, control group; H_2_O_2_, senescent group; GA‐D, GA‐D treatment group; mock‐vehicle, GA‐D treatment group plus empty carrier; h‐14‐3‐3ε, GA‐D treatment group plus *YWHAE* overexpression; sh‐14‐3‐3ε, GA‐D treatment group plus *YWHAE* knockdown; $\bar{x}\pm \mathrm{SD}$, mean ± standard deviation. *p < 0.05, **p < 0.01. CaM, calmodulin; CaMKII, CaM‐dependent protein kinase II; GA‐D, ganoderic acid D; H_2_O_2_, hydrogen peroxide; hAMSC, human amniotic mesenchymal stem cell; Nrf2, nuclear erythroid 2‐related factor 2.

**FIGURE 4:**
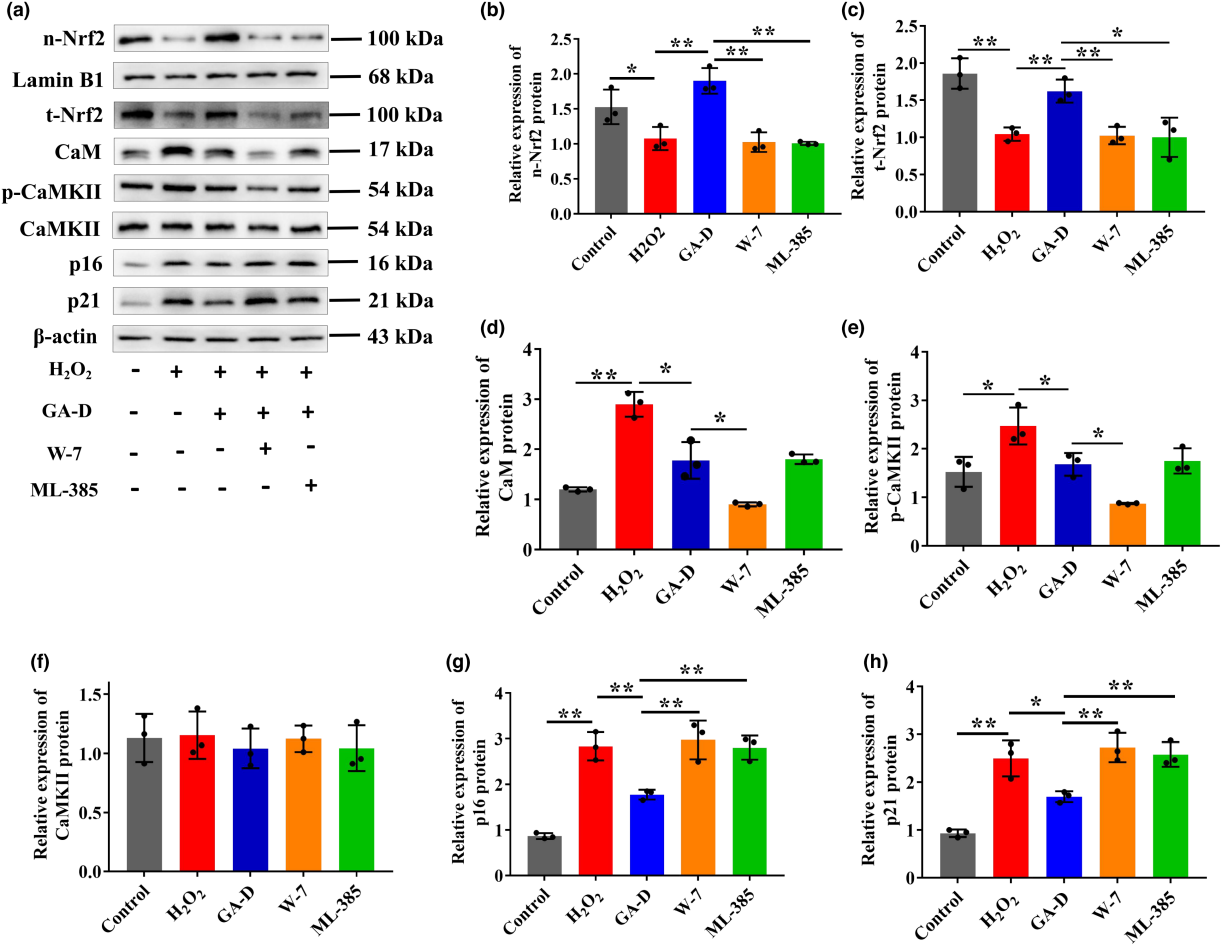
Relationship between CaM/CaMKII and Nrf2/HO‐1/NQO1 signaling pathways. (a) Changes in n‐Nrf2, t‐Nrf2, CaM, p‐CaMKII, CaMKII, p16^INK4a^, and p21 expression in hAMSCs treated with W‐7 (a CaM inhibitor) and ML385 (an Nrf2 inhibitor). (b–h) Relative expression levels of n‐Nrf2, t‐Nrf2, CaM, p‐CaMKII, CaMKII, p16^INK4a^ and p21. N = 3. N‐Nrf2, nuclear Nrf2; t‐Nrf2, total Nrf2; p‐CaMKII, phosphorylated CaMKII; control; control group; H_2_O_2_, senescent group; GA‐D, GA‐D treatment group; W‐7, GA‐D treatment group plus W‐7; ML‐385, GA‐D treatment group plus ML‐385; $\bar{x}\pm \mathrm{SD}$, mean ± standard deviation. *p < 0.05, **p < 0.01. CaM, calmodulin; CaMKII, CaM‐dependent protein kinase II; GA‐D, ganoderic acid D; hAMSC, human amniotic mesenchymal stem cell; Nrf2, nuclear erythroid 2‐related factor 2.

After the knockdown and overexpression of *YWHAE*, we further analyzed the expression levels of CaM and its downstream signaling molecules CaMKII and phosphorylated CaMKII (p‐CaMKII), as well as the components of the Nrf2/HO‐1/NQO1 signaling pathway. We found that these signaling molecules changed when *YWHAE* was overexpressed or knocked down (Figure 3a,b). H_2_O_2_ treatment increased the expression of CaM (Figure 3c) and p‐CaMKII (Figure 3d) but inhibited the intranuclear transfer of Nrf2 (Figure 3f,g). Compared with the H_2_O_2_ group, GA‐D markedly inhibited the protein expression of CaM and p‐CaMKII (Figure 3c–e) and enhanced the intranuclear expression of Nrf2 (Figure 3f,g), as well as its downstream targets such as HO‐1 (Figure 3h) and NQO1 (Figure 3i). However, the expression of these proteins in the sh‐14‐3‐3ε group showed little change compared to that in the H_2_O_2_ group (Figure 3). When *YWHAE* was overexpressed, the changes in the expression of these proteins were not significantly different compared to those in the GA‐D group. These data suggested that the 14‐3‐3ε isoform mediates the anti‐senescent effect of GA‐D on senescent hAMSCs via the CaM/CaMKII and Nrf2/HO‐1/NQO1 pathways. Additionally, cells were treated with the CaM inhibitor W‐7 and the Nrf2 inhibitor ML385 to explore the relationship between the abovementioned signals. W‐7 not only inhibited the protein expression of CaM (Figure 4a,d) and p‐CaMKII (Figure 4a,e) but also decreased the expression levels of total (t‐Nrf2) and nucleus (n‐Nrf2) Nrf2 proteins. However, ML385 caused a sharp decrease in Nrf2 expression (t‐Nrf2 and n‐Nrf2) (Figure 4b,c) but did not affect the expression of CaM and p‐CaMKII. Interestingly, both W‐7 and ML‐385 caused an increase in the primary senescence markers p21 and p16 in the GA‐D group (Figure 4g,h). These data suggest that GA‐D may activate the CaM/CaMKII pathway to facilitate the intranuclear translocation of Nrf2 and finally retard the hAMSCs senescence.

### 2.5 GA‐D prevents d‐gal‐caused aging in mice by enhancing the antioxidative defense capability

**FIGURE 5:**
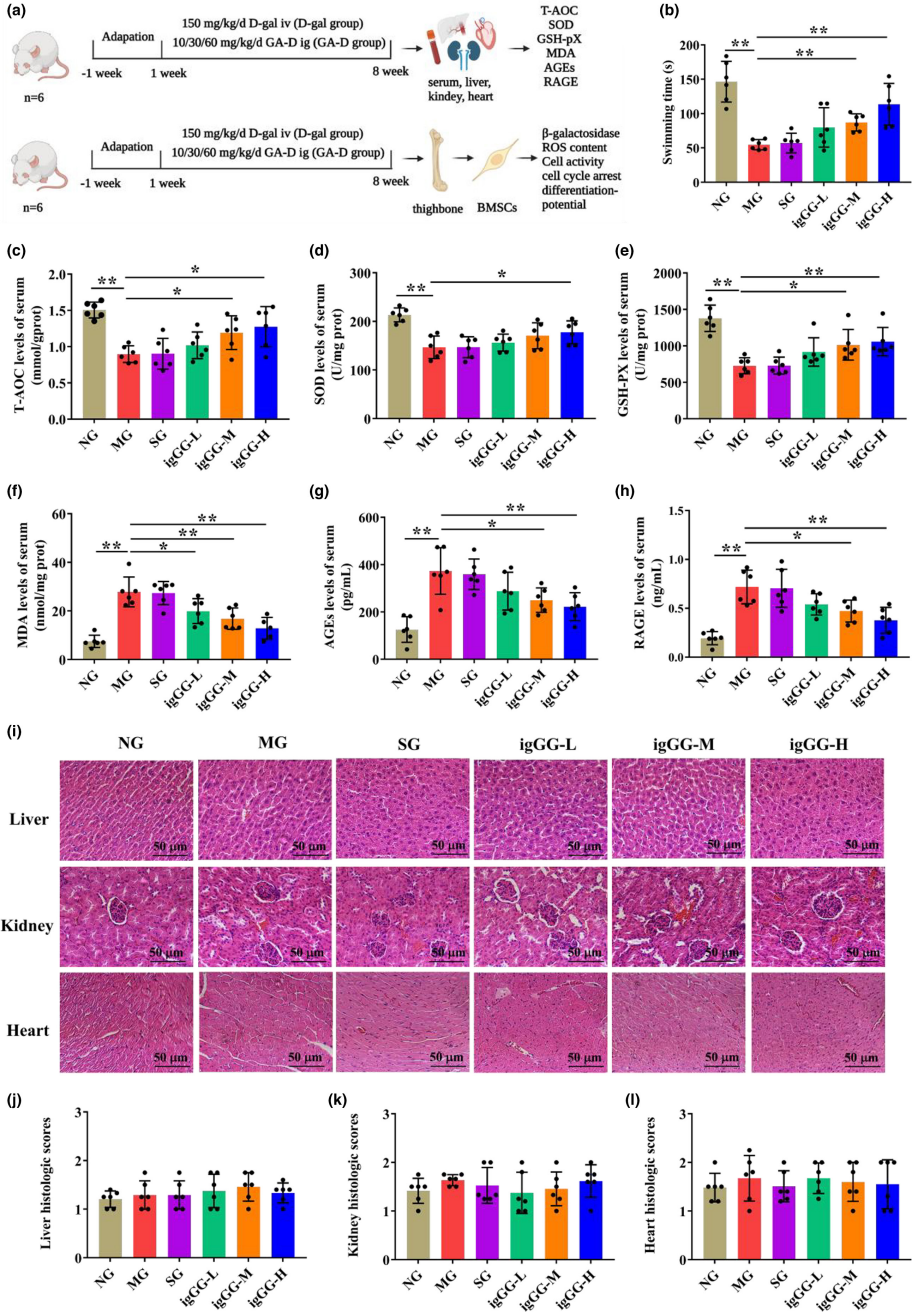
GA‐D enhanced the defense against oxidative stress on the sera in *d‐gal*‐caused aging mice. (a) The experimental design. (b) The swimming test of mice in the different groups. (c) Histopathological organization of the liver, kidney, and heart tissues in different mice groups, analyzed via hematoxylin–eosin (HE) staining. Scale bar: 200 μm. (d–f) The histological scores of liver, kidney and heart tissues in different mouse groups. (g–l) Activity of T‐AOC, SOD, MDA, GSH‐px, AGEs, and RAGE in the sera of mice exposed to different treatments. N = 6. T‐AOC, total antioxidant capacity; SOD, superoxide dismutase; GSH‐px, glutathione peroxidase; MDA, malondialdehyde; AGEs, advanced glycation end products; RAGEs, receptor for advanced glycation end products; NG, normal group; MG, model group (*d‐gal*‐caused aging mice); SG, solvent group (*D‐gal*‐caused aging mice treated with 0.1% DMSO via intragastric administration); igGG‐L, in vivo low‐dose GA‐D treatment group; igGG‐M, in vivo medium‐dose GA‐D treatment group; igGG‐H, in vivo high‐dose GA‐D treatment group; $\bar{x}\pm \mathrm{SD}$, mean ± standard deviation. *p < 0.05, **p < 0.01. GA‐D, ganoderic acid D.

To determine whether GA‐D can exert an anti‐aging effect in vivo, an aging mouse model was subjected to oxidative stress using *d‐gal* (Hsu et al., 2020) (Figure 5a). Firstly, the physical strength of mice in each group was measured via the weight‐loaded forced swimming test (Sun et al., 2020). The swimming time of the mice in the model group (MG) was decreased when compared with the normal group (NG) (Figure 5b). However, the swimming time decline caused by d‐gal was significantly reversed in the in vivo medium‐dose GA‐D treatment group (igGG‐M) and in vivo high‐dose GA‐D treatment group (igGG‐H). Next, GA‐D decreased oxidative stress marker levels in the blood and tissues of aging mice. The levels of total antioxidant capacity (T‐AOC), superoxide dismutase (SOD), and glutathione peroxidase (GSH‐Px) in the sera of mice in the MG group were significantly decreased compared with those of the NG group, but all were increased after three different doses of GA‐D treatment (especially at 60 mg/kg/day) (Figure 5c–e). Conversely, the levels of malondialdehyde (MDA), advanced glycation end products (AGEs), and receptor for advanced glycation end products (RAGEs) significantly increased in the sera of mice in the MG group, but they all decreased after treatment with three different doses of GA‐D (Figure 5f–h). Furthermore, the level of T‐AOC, SOD, and GSH‐Px in the liver, kidney, and heart tissues was markedly lower in the MG group than in the NG group (Figure S5a–l) but was boosted in the in vivo low‐dose GA‐D treatment (igGG‐L), igGG‐M, and igGG‐H groups. MDA levels decreased in each GA‐D group, especially in the igGG‐H group (Figure S5a–l). Additionally, the solvent DMSO did not interfere with the effect of GA‐D (Figure S5a–l). More importantly, there was no pathological damage at the highest GA‐D concentration in the liver, kidney, and heart tissues, indicating that GA‐D was not toxic to the mice (Figure 5i–l). Taken together, these data indicated that GA‐D prevents *d‐gal‐*caused aging in mice by enhancing antioxidative defense capability.

### 2.6 GA‐D prevents premature senescence of BMSCs in d‐gal‐caused aging mice

**FIGURE 6:**
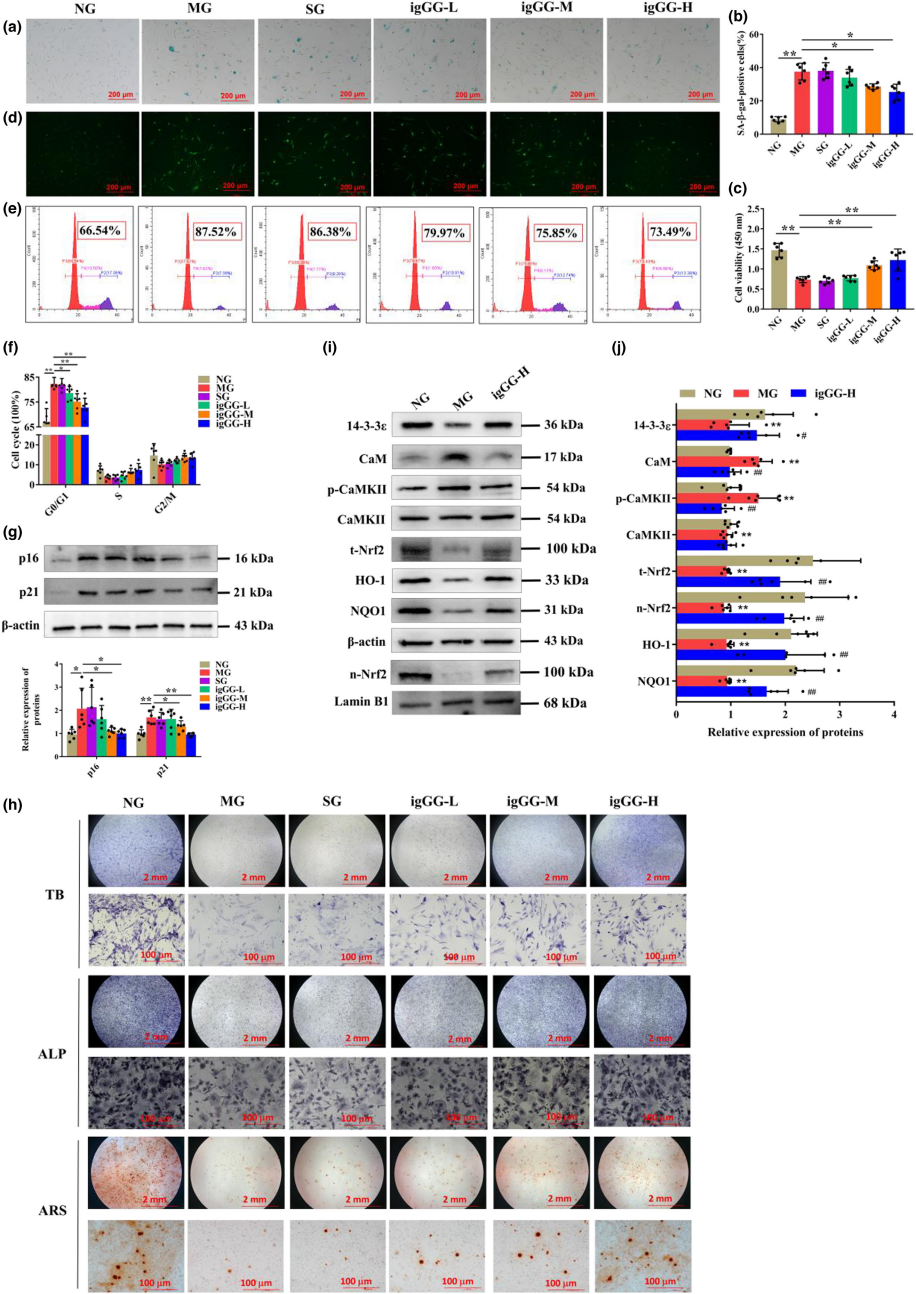
Effect of GA‐D on premature senescence of BMSCs in d‐gal‐caused aging mice. (a) Effect of different concentrations of GA‐D on *β*‐galactosidase production in senescent BMSCs. Scale bar: 200 μm. (b) Percentage of SA‐*β*‐gal‐positive cells. (c) Cell viability of BMSCs determined using CCK8 assay. (d) Intracellular ROS levels in BMSCs were detected after pretreatment of GA‐D. Scale bar: 200 μm. (e) Cell cycle distribution of BMSCs determined via flow cytometry. (f) the number of BMSCs in different stages of the cell cycle distribution. (g) Relative expression levels of p16^INK4a^ and p21. (h) Toluidine blue (TB), alkaline phosphatase (ALP), and alizarin red S (ARS) staining of BMSCs. Scale bar: 2 mm, 100 μm. (i) Changes in 14‐3‐3ε, CaM, p‐CaMKII, CaMKII, t‐Nrf2, n‐Nrf2, HO‐1, and NQO1 expression levels in BMSCs in each group. (j) Relative expression levels of 14‐3‐3ε, CaM, p‐CaMKII, CaMKII, t‐Nrf2, n‐Nrf2, HO‐1, and NQO1 expression in BMSCs in the each groups. N = 6. N‐Nrf2, nuclear Nrf2; t‐Nrf2, total Nrf2; p‐CaMKII, phosphorylated CaMKII; NG, normal group; MG, model group (*d‐gal‐caused* aging mice); SG, solvent group (*d‐gal‐caused* aging mice treated with 0.1% DMSO via intragastric administration); igGG‐L, in vivo low‐dose GA‐D treatment group; igGG‐M, in vivo medium‐dose GA‐D treatment group; igGG‐H, in vivo high‐dose GA‐D treatment group. $\bar{x}\pm \mathrm{SD}$, mean ± standard deviation. *p < 0.05, **p < 0.01. BMSC, bone‐marrow mesenchymal stem cell; CaM, calmodulin; CaMKII, CaM‐dependent protein kinase II; CCK8, Cell Counting Kit‐8; DMSO, dimethyl sulfoxide; GA‐D, ganoderic acid D; ROS, reactive oxygen species.

We further explored the effect of GA‐D on bone‐marrow mesenchymal stem cells (BMSCs) in aging mice. In this study, consistent with the previous reports (Baddoo et al., 2003), the BMSCs isolated from mice in each group expressed surface markers of MSCs, including CD44, CD29, and stem cells antigen‐1 (Sca‐1), whereas those of HSCs, including CD31 and CD117, were not expressed (Figure S6). Notably, we found that BMSCs in *d‐gal‐*caused aging mice showed a senescent state compared with NG group mice, including increased production of *β*‐galactosidase (Figure 6a,b) and intracellular ROS (Figure 6d), as well as decreased cell viability (Figure 6c), G0/G1 cycle phase arrest (Figure 6e,f), increased expression of p21 and p16^INK4a^ proteins (Figure 6g), and reduced differentiation capability (Figure 6h). The generation of *β*‐galactosidase and intracellular ROS in BMSCs was suppressed, and cell viability was increased in the igGG‐M and igGG‐H groups. However, there was no significant effect on BMSC senescence in the igGG‐L group (Figure 6a–d). At the same time, the number of BMSCs at the G0/G1 phase was partially rescued from 82.30 ± 2.62% (MG) to 78.65 ± 2.64%, 75.15 ± 3.21%, and 72.81 ± 3.53% in igGG‐L, igGG‐M, and igGG‐H groups, respectively (Figure 6e,f). The expression of senescence markers, including p16^INK4a^ and p21 in BMSCs in igGG‐L, igGG‐M, and igGG‐H groups, were significantly reduced compared with those in the MG (Figure 6g). In addition, the expression of alkaline phosphatase and glycosaminoglycan and the number of calcium nodules were significantly increased in BMSCs in igGG‐L, igGG‐M, and igGG‐H groups compared with those in the MG group (Figure 6h), indicating that the differentiation ability of senescent BMSCs could be restored upon GA‐D treatment. Further, we explored whether GA‐D delayed BMSCs senescence in vivo via regulating 14‐3‐3ε and CaM/CaMKII/Nrf2 axis. Compared with the NG, d‐gal treatment reduced the expression of 14‐3‐3ε isoform, increased the expression of CaM and p‐CaMKII, and inhibited the intranuclear transfer of Nrf2 in the MG (Figure 6i,j). However, GA‐D markedly increased the 14‐3‐3ε protein expression, inhibited the protein expression of CaM and p‐CaMKII, and enhanced the intranuclear expression of Nrf2 as well as its downstream targets such as HO‐1 and NQO1 in the igGG‐H group (Figure 6i,j). These results suggest that the protective mechanism of GA‐D against BMSCs senescence in vivo is consistent with the protective mechanism against hAMSCs in vitro.

## 3 DISCUSSION

Based on our previous study (Xu et al., 2020), the specific target and underlying mechanism of GA‐D against H_2_O_2_‐induced hAMSC senescence were further demonstrated by targeting 14‐3‐3ε to activate the CaM/CaMKII/Nrf2 signaling pathway in the current study. Furthermore, GA‐D, an anti‐aging candidate compound, exhibited anti‐aging protective effects in a *d‐gal*‐caused aging model mouse, which also retarded the BMSCs senescence in aging mice.

Stem cell senescence is inherently associated with the occurrence of organismal aging and age‐related diseases. Senescent cells can result in tissue degeneration and/or disruption in organisms (De Magalhães & Passos, 2018). Thus far, ever‐increasing data have shown the senescence of tissue‐specific stem cells as an attractive theory for the decline of tissue and organ function during mammalian aging (Cianflone et al., 2020). Hence, age‐related studies based on stem cell senescence models will be beneficial in discovering aging‐associated genes and revealing the targets and the molecular signal regulation network that change stem cell homeostasis caused by aging, which will facilitate the development of treatment strategies for senile diseases.

Reactive oxygen species generated in cells via the mitochondrial respiration chain, including H_2_O_2_, are efficient inducers of oxidative damage and mediators of cellular senescence (Giorgio et al., 2007). It has been reported that the main reason for H_2_O_2_‐induced cell senescence is that H_2_O_2_ treatment can increase intracellular ROS levels and DNA damage by increasing intracellular Ca^2+^ content and interrupting intracellular Ca^2+^ homeostasis (Aiken et al., 1995). Ca^2+^ homeostasis is disrupted in senescent cells, and the 14‐3‐3ε isoform regulates intracellular Ca^2+^ homeostasis by regulating CaM signaling (Russo et al., 2012). In this study, H_2_O_2_ was used to stimulate the senescence of hAMSCs through ROS‐caused DNA damage (Jurk et al., 2012; Menon et al., 2013), whereas exogenous H_2_O_2_ could also facilitate extracellular calcium influx, causing calcium dysregulation (Morad et al., 2021; Wang & Joseph, 2000). Disruption of intracellular Ca^2+^ homeostasis leads to cell senescence (Kim et al., 2020). Therefore, we hypothesized that the anti‐senescent effect of GA‐D may be involved in regulating intracellular ROS and Ca^2+^ levels and restoring calcium homeostasis in H_2_O_2_‐induced hAMSCs senescence. Available evidence shows that CaM is a primary Ca^2+^ sensor that rapidly binds to four Ca^2+^ ions, followed by CaM‐Ca^2+^ binding to CaMKII and causing CaMKII autophosphorylation (Forest et al., 2008; Shifman et al., 2006). Hence, both CaM and CaMKII play major roles in Ca^2+^ signal transduction. Consistent with the previous results (Morad et al., 2021; Wang & Joseph, 2000), H_2_O_2_ resulted in a significant increase in the expression of CaM and p‐CaMKII in hAMSCs. Moreover, our data indicated that GA‐D inhibited the production of intracellular ROS (Figure 2d,e), while the expression of CaM and p‐CaMKII was significantly inhibited in H_2_O_2_‐induced senescent hAMSCs after GA‐D treatment (Figure 3c,d). These data suggest that GA‐D may inhibit H_2_O_2_‐induced Ca^2+^ levels and restore calcium homeostasis in H_2_O_2_‐induced senescent hAMSCs. However, the mechanism of how GA‐D downregulates intracellular calcium levels elevated by H_2_O_2_ induction needs to be elucidated.

Consistent with our previous findings (Xu et al., 2020), GA‐D inhibited the hAMSCs senescence induced by H_2_O_2_ by promoting the expression of Nrf2 (Figure 3g) and its subsequent intranuclear translocation from the cytoplasm to the nucleus (Figure 3f), while inactivation of Nrf2 signaling reversed the anti‐senescent properties of GA‐D (Xu et al., 2020). Moreover, increased nuclear translocation of Nrf2 by GA‐D boosted the protein expression of downstream targets such as HO‐1 (Figure 3h) and NQO1 (Figure 3i), which contributed to the anti‐aging effect. Nrf2 is a master regulator of cellular antioxidative defense and cytoprotective systems. Multiple mechanisms, including the Nrf2‐Keap1 complex formation in the cytoplasm, the Nrf2 degradation via ubiquitination, and the inhibition of Nrf2 nuclear translocation, may inhibit the activation of Nrf2 signaling (Yu & Xiao, 2021; Yuan et al., 2021). Therefore, we attempted to elucidate the underlying mechanism of how GA‐D activates Nrf2 signaling. Our previous study showed that GA‐D activates the signaling molecule of phosphorylated protein kinase R‐like endoplasmic reticulum kinase, subsequently promoting the expression and intranuclear transfer of Nrf2, thereby enhancing the expression of the downstream effector peroxidase III (Xu et al., 2020). However, the available evidence failed to accurately describe the upstream events of Nrf2 activation. In this study, the 14‐3‐3ε isoform may be a vital target for the anti‐senescent effect of GA‐D in hAMSCs. We found that GA‐D was bound to 14‐3‐3ε using the Autodock software (Figure 1e,f). The result of target validation further demonstrated that 14‐3‐3ε is the target protein of GA‐D by both DARTS and CETSA approaches (Figure 1a–d). Due to the easy operation and good compatibility with cell lysate or intact cells, these two protein stability‐based methods, based on different principles, have been widely used over the past 20 years as two major routine methods for drug target discovery (Drewes & Knapp, 2018; Lyu et al., 2020). However, further studies are still required to reveal the binding site of GA‐D on the target 14‐3‐3ε. More importantly, we found that *YWHAE* (14‐3‐3ε‐encoding gene) knockdown reversed the anti‐senescence effect of GA‐D, causing a dramatic increase in the proportion of SA‐*β*‐gal‐positive cells, the proportion of G2/M‐phase cells, intracellular ROS levels, and p16 and p21 expression, as well as a significant decrease in differentiation ability, which was similar to the results obtained for the H_2_O_2_ group (Figure 2). Likewise, *YWHAE* knockdown also reversed the expression and intranuclear transfer of Nrf2, as well as the protein expression of Nrf2 downstream targets such as HO‐1 and NQO1, activated by GA‐D in senescent hAMSCs (Figure 3). Although GA‐D markedly upregulated the expression of 14‐3‐3ε at mRNA and protein levels in senescent hAMSCs (Figures 1g–i), *YWHAE* overexpression did not cause a significant alteration in the anti‐senescent effect of GA‐D, and the expression of the key Nrf2 signaling pathway members. Previous studies showed that 14‐3‐3 proteins exerted a positive and/or negative dual role in controlling lifespan. Overexpression of *YWHAE* extended life span and that this is dependent on FoxO (a forkhead family transcription factor) in *Caenorhabditis elegans*, while loss of 14‐3‐3ɛ protein function could be sufficient to trigger FoxO nuclear localization and extend wild‐type lifespan in *Drosophila*, but overexpression of *YWHAE* did not significantly affect lifespan (Nielsen et al., 2008). In addition, the interaction between all 14‐3‐3ε and FoxO was strongly reduced under oxidative stress, and its inhibitory effect on lifespan was lost (Nielsen et al., 2008). In this study, the senescent hAMSCs model was established by exposure to oxidative stress. However, we did not explore the interaction between 14‐3‐3ε and FoxO in the presence/absence of GA‐D. Accordingly, the above findings suggest that the 14‐3‐3ε isoform is an important target of GA‐D and plays a positive role in the anti‐senescent effect of GA‐D in senescent hAMSCs. However, the underlying mechanism by which GA‐D upregulates the expression level of 14‐3‐3ε requires further investigation.

As mentioned above, the regulatory effect of GA‐D on intracellular Ca^2+^ levels and calcium homeostasis remains to be elucidated. Interestingly, further investigation showed that *YWHAE* knockdown reversed the inhibitory state of CaM and p‐CaMKII expression caused by GA‐D in the senescent hAMSCs, and the expression levels of these signaling molecules were restored in the sh‐14‐3‐3ε group, and reached the levels of the H_2_O_2_‐induced group (Figure 3c,d). 14‐3‐3ε isoform, a mitochondrial import‐stimulation factor, is encoded by *YWHAE* on chromosome 17 in humans and is highly expressed in mammalian cells (Aghazadeh & Papadopoulos, 2016). Moreover, the human 14‐3‐3ε isoform can interact directly with human CaM and involve in cell proliferation and signaling transduction (Luk et al., 1999). Inhibitory interactions exist between 14‐3‐3ε isoform and certain Ca^2+^ pump isoforms in the cytoplasmic membrane of CHO and HeLa cells (Linde et al., 2008; Rimessi et al., 2005). Therefore, these data suggest that the 14‐3‐3ε isoform modulates intracellular Ca^2+^ homeostasis through the interaction between CaM or the plasma membrane Ca^2+^ pump and 14‐3‐3ε. Most proteins do not function as isolated entities but rather by forming a dynamic complex with other proteins in a specific environment. The dynamic physical complex formed upon PPIs is essential for the regulation of biological processes and diseases (Stevers et al., 2018). Therefore, discovering appropriate modulators (inhibitors and/or stabilizers) of such a dynamic physical complex is an attractive concept for disease intervention. As one of the most important hub proteins, 14‐3‐3 proteins participate in a widespread physiological process through PPIs with several partner proteins. Small‐molecule modulators, both inhibitors and stabilizers, of 14‐3‐3 PPIs have been extensively and intensively investigated via high‐throughput and in silico screening (Hartman & Hirsch, 2017; Ottmann, 2013). For example, the activity of CaMK kinase 2 (CaMKK2), an upstream molecule of the CaMK signaling cascade, is suppressed by phosphorylation in a process that involves binding to the 14‐3‐3γ, thereby maintaining CaMKK2 in a phosphorylation‐mediated inhibitory state (Santo et al., 2020). Fusicoccin A, a fungus‐derived diterpene glycoside, may stabilize the PPIs between 14‐3‐3γ and phosphorylated CaMKK2 and increase the stability of CaMKK2, which causes a decrease in the 14‐3‐3γ‐mediated dephosphorylation of CaMKK2 (Santo et al., 2020). In addition, fusicoccin A promotes platelet aggregation via the interaction between 14‐3‐3 and adhesion receptor glycoprotein Ib‐IX‐V (Camoni et al., 2011), and stabilizes the H+‐ATPase and 14‐3‐3 complex in plants (Camoni et al., 2013). Although small‐molecule compounds are employed to regulate 14‐3‐3 PPIs, the modulator of the 14‐3‐3ε isoform remains unknown. Our results showed that GA‐D, a fungus‐derived triterpene, targeted the 14‐3‐3ε isoform to prevent hAMSC senescence through calcium signaling involving CaM and CaMKII phosphorylation. Therefore, the regulation of 14‐3‐3ε interaction with CaM, phosphorylated CaMKII, or other proteins by GA‐D is worthy of further exploration.

Here, we preliminarily demonstrated that GA‐D retarded the hAMSCs senescence by targeting the 14‐3‐3ε isoform to activate the CaM/CaMKII and Nrf2/HO‐1/NQO1 signaling pathways. Our data showed that *YWHAE* knockdown downregulated the expression and intranuclear translocation of Nrf2 (Figure 3f,g) and expression of its downstream target proteins HO‐1 and NQO1 (Figure 3h,i). *YWHAE* knockdown also upregulated the expression of CaM and phosphorylated CaMKII (Figure 3c,d) in senescent hAMSCs after GA‐D treatment, and the expression levels of these signaling molecules activated by GA‐D were similar to those in the H_2_O_2_‐induced group. In contrast, changes in the expression of these signaling molecules upon *YWHAE* overexpression were stronger than in the GA‐D treatment group (Figure 3). In line with these findings, 14‐3‐3ε was identified as the upstream effector of the CaM/CaMKII and Nrf2/HO‐1/NQO1 pathways. Additionally, we found that W‐7 (a CaM inhibitor) not only suppressed the expression of CaM and p‐CaMKII (Figure 4d,e) but also inhibited the expression and intranuclear transfer of Nrf2 (Figure 4b,c). The expression and intranuclear transfer of Nrf2 were restrained in the presence of ML385 (an Nrf2 inhibitor); no effects were observed on the expression of CaM and p‐CaMKII after ML385 treatment in the ML385 group compared to the GA‐D group (Figure 4a). Furthermore, the expression and intranuclear transfer of Nrf2 showed essentially the same results regardless of ML385 or W‐7 treatment. These findings suggested that CaM/CaMKII are the upstream effectors of the Nrf2/HO‐1/NQO1 pathway. Therefore, we speculated that GA‐D might stabilize the PPIs between 14‐3‐3ε and CaM or phosphorylated CaMKII, which maintains calcium homeostasis and promotes intranuclear transfer of Nrf2, as well as HO‐1 and NQO1 expression, to prevent hAMSC senescence. However, this requires further evidence.

Although our results have demonstrated that GA‐D exerts a potent anti‐senescence effect against the stem cell senescence model (Xu et al., 2020), these data cannot explain whether GA‐D exerts anti‐aging effects in vivo. This study determined the anti‐aging effect of GA‐D in a *d‐gal‐*caused aging murine model. *d‐gal* establishes a mimetic aging model based on the metabolic and free radical theory of aging. The *d‐gal* accumulation leads to osmotic imbalance and harmful oxidative stress to cells, causing aging‐like changes or symptoms in mice. Thus, *d‐gal‐*caused aging mice have been extensively employed in anti‐aging studies (Xiao et al., 2012). Consistent with the previous findings (Rusu et al., 2020), long‐term injection of *d*‐gal reduced the activity of T‐AOC, GSH‐Px, and SOD and increased the content of MDA, AGEs, and RAGEs in the blood, kidney, liver, and heart (Figures 5g–l; Figure S4), indicating that the accelerated aging model was successfully established. GA‐D enhanced the activity of T‐AOC, GSH‐Px, and SOD and decreased the content of MDA, AGEs, and RAGEs in different tissues and organs, indicating that GA‐D exerted anti‐aging effects by inhibiting the oxidative stress responses in d‐gal‐caused aging mice. Of note, stem cell senescence and depletion are important causes of aging based on the theory of stem cell senescence (Cianflone et al., 2020; Díaz‐Morenoa et al., 2018; Vyver et al., 2021). Furthermore, some studies showed that d‐gal induces senescence of HSCs and regression of bone marrow and HSCs, which are similar to the symptoms of aging (Boyette & Tuan, 2014; Li et al., 2016). Hence, we further investigated whether BMSCs were senescent in *d‐gal‐*caused aging mice and the effect of GA‐D on BMSCs. Interestingly, the cell viability and differentiation potential of BMSCs were reduced, while the intracellular ROS levels were increased. Furthermore, the proportion of arrested cells at the G0/G1 phase and SA‐*β*‐gal‐positive cells in BMSCs were elevated in d‐gal‐caused aging mice (Figure 6), indicating that BMSCs were senescent. More importantly, we found that GA‐D treatment could improve these aging properties, including the production of SA‐*β*‐gal‐positive cells, reduced cell viability, ROS formation, cell cycle arrest, and decreased differentiation potential (Figure 6a–h), and retard the senescence of BMSCs in d‐gal‐caused aging mice. In addition, consistent with the protective in vitro mechanism against hAMSCs senescence, GA‐D retarded BMSCs senescence in *d‐gal‐*caused aging mice through increasing the 14‐3‐3ε protein expression, inhibiting the protein expression of CaM and p‐CaMKII and enhancing the intranuclear expression of Nrf2, as well as its downstream targets such as HO‐1 and NQO1 (Figure 6i,j). Therefore, our results suggest that GA‐D may be a promising candidate for the development of anti‐aging agents.

In addition, cellular senescence can be triggered by various intrinsic and extrinsic stimuli, as well as developmental signals, whereby the types of senescence mainly include replicative senescence (RS), oncogene‐induced senescence (OIS), stress‐induced premature senescence (SIPS), and developmental senescence (DS) (Mylonas & O'Loghlen, 2022; Zhou et al., 2020). In this study, GA‐D showed significant protective effects against MSCs senescence induced by oxidative stressors H_2_O_2_ and d‐gal via the same signal pathway. To the senescence induced by other oxidative stressors such as doxorubicin (Bashiri Dezfouli et al., 2020; Maejima et al., 2008), GA‐D may show a protective effect as it can reduce oxidative stress‐induced increases in SA‐*β*‐gal‐positive cells and senescent cell markers such as p21 and p16. However, it remains unclear whether GA‐D can show protective effects against senescence induced by other stressors, including RS, OIS, DS, and other SIPS. The effect of GA‐D on senescence induced by other stressors needs to be explored in our future research.

In conclusion, our results demonstrated that GA‐D protects hAMSCs against H_2_O_2_‐induced senescence by targeting 14‐3‐3ε to activate the CaM/CaMKII and Nrf2/HO‐1/NQO1 signaling pathways (Figure S7). Furthermore, GA‐D showed a consistent anti‐aging effect in vivo, especially impeding the senescence of BMSCs in d‐gal‐caused aging mice via the same protective mechanism as that in vitro. These results suggest that the H_2_O_2_‐induced stem cell senescence model might be an effective initial screening model for anti‐aging agents, and GA‐D may be a potential anti‐aging agent. This study provides a scientific basis for the development of *G. lucidum* in anti‐aging agents and presents scientific evidence for the theory of stem cell senescence. However, the anti‐aging mechanism of GA‐D requires a further in‐depth study.

## 4 EXPERIMENTAL PROCEDURES

### 4.1 The source of natural small‐molecule GA‐D

GA‐D was obtained from a Biotechnology Company (Chengguang Biot. Co. Ltd). Its physical–chemical properties, including chemical purity, molecular weight, and chemical structure, are available from our previous study (Xu et al., 2020).

### 4.2 Animal experiments

ICR male mice (18–22 g) obtained from the Tianqin Biological Technology Limited Company (Certification No: SCXK [Xiang] 2019‐0013), were pair‐housed in plastic cages in our animal breeding room and exposed to a 12/12 h light/dark cycle. The temperature of the animal breeding room was maintained at ca. 23°C. The mice were given food and fresh water ad libitum. The animal experiments were approved by the Ethics Committee of Experimental Animals of Zunyi Medical University (permit number: Lunshen [2019]‐1‐173). All procedures involving mice studies conformed to the ethical principles of animal welfare.

### 4.3 Experimental groups and drug treatment

An aging mouse model was generated via subcutaneous (s.c) injection of 150 mg/kg/day d‐galactose (Sigma) for 8 weeks and treatment with GA‐D and DMSO (Sigma). The animals were randomly assigned to one of the following six groups (n = 12 mice/group): (1) NG, mice treated with normal saline (s.c) as a vehicle; (2) MG, d‐gal 150 mg/kg/day (s.c.) + normal saline (intragastric administration, i.g.); (3) solvent control group (SG), d‐gal 150 mg/kg/day (s.c.) + DMSO (0.1%) (i.g); (4) low‐dose GA‐D group (igGG‐L), d‐gal 150 mg/kg/day (s.c.) + GA‐D 10 mg/kg/day (i.g); (5) med‐dose GA‐D group (igGG‐M), d‐gal 150 mg/kg/day (s.c.) + GA‐D 30 mg/kg/day (i.g); (6) high‐dose GA‐D group (igGG‐H), d‐gal 150 mg/kg/day (s.c.) + GA‐D 60 mg/kg/day (i.g). The mice were continuously treated once daily for 8 weeks.

### 4.4 Cell isolation culture

Following a previously described method (Wang et al., 2020), hAMSCs were isolated from the placental amnion of healthy pregnant women, using collagenase type II (Solarbio) and deoxyribonuclease I (Solarbio) after obtaining informed consent. According to the whole bone‐marrow culture methods described by Yang et al. (2017), BMSCs were isolated from mice treated as described in Section 4.3. The hAMSCs and BMSCs were maintained at 37°C in a 5% (v/v) CO_2_ incubator (Forma 3110; Thermo) with a humidified atmosphere. Their culture medium consisted of low‐glucose Dulbecco's modified Eagle's medium (LG‐DMEM; Gibco), 10% fetal bovine serum (FBS; Gibco), 1% non‐essential amino acids (Gibco), 1% l‐alanyl‐glutamine dipeptide (l‐GlutaMAX) (Gibco), and 10 ng/ml basic fibroblast growth factor (Peprotech). The culture medium was refreshed every 3 days. The growth of the cells was observed under an inverted microscope (Olympus). When the cells reached about approximately 80% confluency, they were passaged using 0.125% trypsin (including 0.02% EDTA‐2Na) (Sangon Biotech). BMSCs and hAMSCs at passages 2 (P2) and 3 (P3), respectively, were used for further experiments.

### 4.5 Flow cytometry analysis

The BD Stemflow Human MSC analysis kit (Cat. No. 562245; BD Biosciences) and the OriCell Mice MSC analysis kit (Cat. MUXMX‐09011; Oricell) were employed to detect the surface markers of hAMSCs and BMSCs. P3 hAMSCs and P2 BMSCs were trypsinized, collected, and washed twice with Dulbecco's phosphate‐buffered saline (D‐PBS). The hAMSCs were then re‐suspended and adjusted to a density of 1 × 10^6^ cells/ml in 0.1% bovine serum albumin (BSA). Fluorescence‐labeled antibodies were separately added to each tube and incubated with the cells in the dark for 1 h. After washing with D‐PBS, the cells were centrifuged at 250 *g* at 4°C for 5 min, and then, the supernatant was discarded and fixed with 200 μl of 1% paraformaldehyde (PFA), vortexed and blended. Finally, flow cytometry (BD Biosciences) was used to analyze the labeled cells. The corresponding fluorescence‐labeled non‐immune isotype IgG antibodies (BD Biosciences) were used as controls. The BMSCs were then re‐suspended and adjusted to a density of 3 × 10^5^ cells/ml in 0.1% BSA. Antibodies were added separately to each tube, incubated with the cells for 30 min, and washed with D‐PBS. The cells were centrifuged at 250 *g* at 4°C for 5 min and then incubated with FITC/PE fluorescently labeled secondary antibody for 30 min. After washing with D‐PBS, the cells were centrifuged at 250 *g* at 4°C for 5 min. Then, the supernatant was discarded and fixed with 200 μl of 0.1% BSA. Flow cytometry (BD Biosciences) was used to analyze the labeled cells. The corresponding fluorescence‐labeled non‐immune isotype IgG antibodies (Oricell) were used as controls. Synchronously, immunocytochemical staining was used to detect the expression of vimentin (mouse anti‐human, 1:100; Gene Tech) and cytokeratin 19 (mouse anti‐human, 1:100; Gene Tech). The hAMSCs were fixed with 4% PFA for 15 min. The cells were permeabilized by exposure to 0.3% Triton X‐100 for 30 min, followed by washing twice with D‐PBS. After blocking nonspecific binding with 5% BSA for 30 min, the cells were incubated overnight at 4°C with primary antibodies. The next day, after washing with D‐PBS three times, the secondary antibody (goat anti‐mouse, 1:4000; Abcam) was added to the cells and then incubated at 37°C for 30 min. The color was developed using diaminobenzidine for 30 s. The nuclei were counterstained with hematoxylin. Finally, a Nikon Eclipse Ti‐S inverted fluorescence microscope (Nikon) was used to observe and image the cells.

### 4.6 Overexpression and knockdown of YWHAE

Recombinant adenovirus expression vectors, including HBAD‐GFP, HBAD ‐Adeasy‐h‐YWHAE‐3xflag‐EGFP, HBAD‐Adeasy‐h‐YWHAE‐shRNA1‐EGFP, HBA ‐D‐Adeasy‐h‐YWHAE‐shRNA2‐EGFP, and HBAD‐Adeasy‐h‐YWHAE‐shRNA‐EGF‐P, were obtained from Shanghai Hanheng Biotechnology Limited Company. The P2 hAMSCs were seeded in six‐well plates at a density of 2 × 10^5^ cells (Corning). The virus was added to the cells in the logarithmic growth phase. Viruses with MOI values of 10, 30, 50, 100, 200, and 300 were used to incubate cells for 8 h before the medium was replaced. Subsequently, green fluorescent protein expression was measured after 72 h. The optimal MOI was selected when fluorescence reached 80%, and virus quantity was at its minimum. P2 hAMSCs were transfected with different viruses and divided into different groups, including the normal control group (control, no virus solution), null‐vector negative control group (mock‐vehicle, transfected with no‐load virus, HBAD‐GFP), 14‐3‐3ε overexpression group (h‐14‐3‐3ε, transfected with HBAD‐Adeasy‐h‐YWHAE‐3xflag‐EGFP), and 14‐3‐3ε knockdown group (sh‐14‐3‐3ε, transfected with HBAD‐Adeasy‐h‐YWHAE‐3‐xflag‐EGFP). After 8 h, the virus‐containing medium was changed with the normal medium, and protein extraction was performed at 24, 48, and 72 h after induction. WB was then used to analyze the levels of the 14‐3‐3ε isoform after the overexpression and knockdown of *YWHAE*.

### 4.7 Cell treatment

P3 hAMSCs with a density of 50% were pretreated with 10 μM GA‐D. After 6 h, hAMSCs were treated with 200 μM H_2_O_2_ for 2 h. The hAMSCs were washed with D‐PBS and then incubated with fresh medium at 37°C. Simultaneously, they were treated with the viral vector for 24 h and then added with GA‐D. DMSO at a concentration of 0.01% was used to dissolve GA‐D. W‐7 and ML385 were dissolved in DMSO and diluted with D‐PBS to concentrations of 25 and 10 mM, respectively, and they were incubated for 1 h before GA‐D treatment. hAMSCs were divided into the following six groups: (1) control group (Control), no virus solution and treatment; (2) model group (H_2_O_2_), 200 μM H_2_O_2_; (3) drug treatment group (GA‐D), 10 μM GA‐D + 200 μM H_2_O_2_; (4) null‐vector negative control group (Mock‐vehicle), HBAD‐GFP + 10 μM GA‐D + 200 μM H_2_O_2_; (5) 14‐3‐3ε overexpression group (h‐14‐3‐3ε), HBAD‐Adeasy‐h‐YWHAE‐3xflag‐EGFP + 10 μM GA‐D + 200 μM H_2_O_2_; (6) 14‐3‐3ε knockdown group (sh‐14‐3‐3ε), HBAD‐Adeasy‐h‐YWHAE‐3xflag‐EGFP +10 μM GA‐D + 200 μM H_2_O_2_.

### 4.8 β‐galactosidase staining

The production of the cell senescence marker *β*‐galactosidase was measured using the *β*‐galactosidase staining kit (Beyotime). The *β*‐galactosidase working solution was prepared according to the manufacturer's instructions. hAMSCs and BMSCs were washed once with D‐PBS and fixed for 15 min with 4% PFA. Then, they were washed three times with D‐PBS and stained with a *β*‐galactosidase working solution for 4 h at 37°C.

### 4.9 Intracellular ROS assay

The intracellular ROS level for hAMSCs and BMSCs was measured using the ROS detection kit from Applygen (red fluorescence) and Beyotime (green fluorescence), respectively. The cells were washed with D‐PBS after incubation with dihydroethidium for 30 min at 24–28°C. Finally, red and green fluorescent protein expression was measured using Nikon Eclipse Ti‐S inverted fluorescence microscope (Nikon) and flow cytometry (BD Biosciences).

### 4.10 The differentiation of hAMSCs and BMSCs

P2 hAMSCs and P1 BMSCs were seeded in 12‐well plates at a density of 2 × 10^4^ with LG‐DMEM culture medium and incubated at 37°C for 24 h. hAMSCs were treated as mentioned in Section 4.7, and BMSCs were isolated from mice treated as described in Section 4.3. High sugar medium (HG‐DMEM), containing 50 mg/L vitamin C (Solarbio), 100 nmol/L dexamethasones (Sigma), and 10% FBS, was used to prepare osteogenic induction medium with 10 mmol/L *β*‐glycerophosphate (Solarbio) and cartilage induction medium with 10 ng/L transforming growth factor‐β3 (Peprotech). They were used to incubate hAMSCs and BMSCs at room temperature. Toluidine blue staining was used to observe chondrogenesis after 5 days. Osteogenic differentiation was preliminarily observed using alkaline phosphatase and alizarin red staining after 7 and 21 days, respectively. The hAMSCs were cultured in adipogenic induction medium (Procell Life Science & Technology Co., Ltd) for 26 days, and oil red O staining was used to observe the formation of lipid droplets.

### 4.11 Cell viability assay

Cell Counting Kit‐8 (CCK8) Cell assay (ab228554; Abcam) was used to measure the BMSCs viability. BMSCs were incubated with CCK8 solution at 37°C for 4 h. A microplate reader (Bio‐Rad) was used to measure the absorbance at 450 nm.

### 4.12 Cell cycle analysis

BMSCs and hAMSCs at different cell cycle stages were detected via the DNA content quantitation assay kit (Solarbio). They were digested with trypsin, collected, and then blended with pre‐cooled 70% ethanol and incubated overnight. The cells were washed with D‐PBS, then mixed with RNase A, and incubated at 37°C for 30 min. Subsequently, the cells were mixed with propidium iodide staining solution and incubated in darkness at 4°C for 30 min. Finally, flow cytometry (BD Biosciences) was used for analysis.

### 4.13 The combination of GA‐D and human 14‐3‐3ε protein through autodock

Autodock Vina 1.1.2 software was used to analyze the binding of GA‐D and human 14‐3‐3ε. The three‐dimensional structure (PDB ID: 2BR9) of human 14‐3‐3ε was obtained from the protein database (http://www.rcsb.org/pdb/home/home.do). ChemBioDraw Ultra 14.0 and ChemBio3D Ultra 14.0 software from CambridgeSoft Corp (PerkinElmer) were used to draw the three‐dimensional structure of GA‐D. Autodock tools were used to convert the structures of small molecules and proteins into pdbqt files with central coordinates (center_x:‐18.1999, center_YRV 3.771, center_z:15.771), and the search space was as follows: size_x: 50; size_y: 50; and size_z: 50. PyMoL 2.3.0 and Ligplotv 2.2.0 were used to analyze the interaction mode of the best scoring posture, as judged using the Vina docking score.

### 4.14 DARTS assay

The DARTS assay was used to detect the binding of GA‐D and the human 14‐3‐3ε protein. P3 hAMSCs were treated as described in Section 4.7. The total protein from hAMSCs was extracted with radio‐immunoprecipitation assay (RIPA) lysis buffer (Solarbio) and quantified with BCA Protein Assay Kit (Beyotime). All the steps were performed on ice. Samples were warmed to 40°C for 10 min and digested enzymatically with pronase E (Yuanye) (concentration of *Streptomyces* proteinase was 5 mg/ml, and the mass ratio of enzyme to protein was 1:75) at 40°C for 15 min. Subsequently, sodium dodecyl sulfate‐polyacrylamide gel electrophoresis (SDS‐PAGE) buffer was added immediately to stop the enzymatic hydrolysis at 100°C for 10 min. The resulting mixtures were separated via SDS‐PAGE and stained with Coomassie blue. WB was used to detect the 14‐3‐3ε level.

For target protein identification, the total protein samples were incubated with 5, 10, 20, and 30 μM GA‐D at room temperature for 80 min, then warmed to 40°C for 10 min, and digested enzymatically with pronase E (concentration of *Streptomyces* proteinase was 5 mg/ml, and the mass ratio of enzyme to protein was 1:25, 1:75, and 1:150) at 40°C for 15 min. Then, SDS‐PAGE buffer was added immediately to stop the enzymatic hydrolysis at 100°C for 10 min. WB was used to detect the 14‐3‐3ε level.

### 4.15 Cellular thermal shift assay

The total protein from P3 hAMSCs was extracted with RIPA lysis buffer (Solarbio) and quantified with BCA Protein Assay Kit (Beyotime). All the steps were performed on ice or at 4°C. The samples were incubated with GA‐D or DMSO at room temperature for 80 min, 100 μl of each cell suspension was then dispensed into PCR tubes and heated at 30–100°C by a thermal cycle for 10 min. Subsequently, SDS‐PAGE buffer was added immediately to stop the enzymatic hydrolysis at 100°C for 10 min. Finally, these supernatants were analyzed by WB.

### 4.16 Weight‐loaded swimming test

The mice were administrated GA‐D for the last time, and the next day, aluminum load (7% of their body weight) was attached to their tails and placed individually into a cylindrical swimming pool (35 cm tall and 40 cm radius) filled with fresh water (23–25°C) to a depth of 35 cm so that mice could not support themselves by touching the bottom with their tails. Then, the time that the mice were unable to bail out the water surface for 5 s was recorded.

### 4.17 Serum and tissue homogenate preparation

The mice were administrated for the last time, and the next day, they were deeply anesthetized with 1% pentobarbital sodium. Blood samples were collected by removing the eyeball, placed at 24–28°C for 30 min, and then centrifuged at 6000 *g* at 4°C for 10 min. Serum samples were separated and stored at −80°C. Subsequently, the above mice were sacrificed by cervical dislocation, and the liver, kidney, and heart tissues were rapidly excised. The tissues were ground in pre‐cooled saline and were centrifuged at 6000 *g* at 4°C for 10 min, and the supernatants were separated and stored at −80°C for further analysis.

### 4.18 Biochemical analysis of serum and tissue of aging mice

Commercial detection kits (Nanjing Jiancheng Bioengineering) were used to detect the activities of T‐AOC, SOD, MDA, and GSH‐Px in the serum, liver, kidney, and heart, according to the manufacturer's protocols. AGE and RAGE detection kits (Jianglai Biology) were used to detect the activity of AGEs and RAGEs in the serum according to the manufacturer's instructions. The protein content in the liver, kidney, and heart homogenates was measured using the BCA Protein Assay Kit (Beyotime).

### 4.19 Hematoxylin and eosin staining

After dissecting the mice, the heart, liver, and kidney tissues were excised and fixed with fresh 4% PFA at 4°C overnight. The tissues were sent to the Department of Pathology at the Affiliated Hospital of Zunyi Medical University for sectioning and hematoxylin and eosin staining. Finally, a Nikon Eclipse Ti‐S inverted fluorescence microscope (Nikon) was used to analyze the stained sections. Based on the scoring standard described by Ferrer et al. (1998) and Hou et al. (2009), the histological slides were blindly read and scored using a scale of 1–4 by two experienced pathologists.

### 4.20 Quantitative reverse transcription‐polymerase chain reaction analysis

Total RNA from hAMSCs was extracted with RNAiso Plus (Takara) and used to synthesize cDNA with the PrimeScript™ RT reagent kit (Takara). PCR was performed and monitored with the SYBR® Premix Ex Taq II (Takara) quantitative reverse transcription‐polymerase chain reaction system, and *actb* was used as the loading control. The relative expression levels of different genes were calculated using the 2^−∆∆Ct^ method. The primers for all genes tested are given in Table S1.

### 4.21 Western blotting

The total protein from hAMSCs and BMSCs was extracted with RIPA lysis buffer and quantified with BCA Protein Assay Kit (Beyotime). Proteins were separated using SDS‐PAGE and subsequently electrotransferred onto polyvinylidene fluoride membranes. The antibodies used were as follows: Anti‐p16 antibody (Abcam; ab51243), anti‐p21 antibody (Abcam; ab109520 and Proteintech; 28248‐1‐AP), anti‐Nrf2 antibody (Abcam; ab62352), anti‐HO‐1 antibody (Huabio), anti‐NQO1 antibody (Huabio), anti‐14‐3‐3ε antibody (Huabio), anti‐calmodulin (CaM) antibody (Abcam; ab45689), anti‐CaMKII antibody (Abcam; ab52476), anti‐CaMKII (phospho T286) antibody (Abcam; ab5683), and anti‐Lamin B (Abcam; ab133741). TBST was used to dilute these antibodies. Subsequently, the membranes were incubated with a horseradish peroxidase‐conjugated secondary antibody (Protein Tech; SA00001‐2) for 120 min at 24–28°C. Finally, the enhanced chemiluminescence hypersensitive luminescent solution (Beyotime) was used to treat the membranes, which were imaged using the Chemi DocTM MP Imaging System (Bio‐Rad). ImageJ software was used to analyze the intensity of the protein bands.

### 4.22 Statistical analysis

All experiments were performed at least thrice, and the data are expressed as the mean ± standard deviation. SPSS Statistics for Windows (version 19.0; SPSS, Inc.) was used to statistically analyze the data. The significance among groups was determined using one‐way analysis of variance and Tukey's test. Statistical significance was set at *p* < 0.05. Graphs were prepared using GraphPad Prism software (version 6.0; GraphPad Software, Inc.).

## AUTHOR CONTRIBUTIONS

Jian‐Hui Xiao contributed to the conceptualization, supervision, critical revision, and financial support for this study. Huan Yuan carried out the experiment, analyzed the data, and wrote the original draft. Jia‐Rong Zhang and Xin‐Xin Zhu participated in the supplementary experiments. Yan Xu and Yi Luo designed the experiments and revised the manuscript. All authors reviewed and approved the final manuscript.

## FUNDING INFORMATION

Science and Technology Foundation of Zunyi Science and Technology Bureau, Grant/Award Number: ZSKH‐HZ‐2020‐222 and ZSK‐RC‐2020‐1; Department of Science and Technology of Guizhou Province, Grant/Award Number: QKHJC‐ZK‐2021‐ZD‐026; Guizhou High‐Level Innovative Talent Support Program, Grant/Award Number: QKHPT‐RC‐GCC[2022]001‐1; Minstry of Science and Technology of People's Republic of China, Grant/Award Number: GKFZ‐2018‐29; National Natural Science Foundation of China, Grant/Award Number: 31960191

## CONFLICT OF INTEREST

The authors declare no competing interests.

## Supporting information


Figure S1
Figure S2
Figure S3
Figure S4
Figure S5
Figure S6
Figure S7

Table S1

## Data Availability

The data of this study are presented in the figures ang tables. Also, the data that support the findings of this study are available from the corresponding author upon reasonable request.
