# Corrigendum to: Ganoderic acid D prevents oxidative stress‐induced senescence by targeting 14‐3‐3ε to activate CaM/CaMKII/NRF2 signaling pathway in mesenchymal stem cells

**DOI:** 10.1111/acel.13732

**Published:** 2022-10-19

Huan Yuan, Yan Xu, Yi Luo, Jia‐Rong Zhang, Xin‐Xin Zhu, Jian‐Hui Xiao, *Aging Cell*, 2022, 21(9):e13686. https://doi.org/10.1111/acel.13686

In the published version of Yuan et al. (2022), the authors noticed the following errors.

In the Discussion, “Figure S4” should have been “Figure S5” and the revised sentence should read as follows:

“Consistent with the previous findings (Rusu et al., 2020), long‐term injection of d‐gal reduced the activity of T‐AOC, GSH‐Px, and SOD and increased the content of MDA, AGEs, and RAGEs in the blood, kidney, liver, and heart (Figure 5g–l; **Figure S5**), indicating that the accelerated aging model was successfully established.”

In Figure 3 legend, p16^INK4a^ and p21 appeared incorrectly in the descriptions for parts (a) and (b–k). The revised caption is given below.

**FIGURE 3** CaM/CaMKII and Nrf2/HO‐1/NQO1 signaling pathways were attenuated after *YWHAE* knockdown. (a) Changes in 14‐3‐3ε, t‐Nrf2, n‐Nrf2, HO‐1, NQO1, CaM, p‐CaMKII, and CaMKII expression in hAMSCs upon different treatments. (b–k) Relative expression levels of 14‐3‐3ε, n‐Nrf2, t‐Nrf2, CaM, CaMKII, p‐CaMKII, HO‐1, and NQO1. *n* = 3. n‐Nrf2, nuclear Nrf2; t‐Nrf2, total Nrf2; p‐CaMKII, phosphorylated CaMKII; Control, control group; H_2_O_2_, senescent group; GA‐D, GA‐D treatment group; Mock‐vehicle, GA‐D treatment group plus empty carrier; h‐14‐3‐3ε, GA‐D treatment group plus *YWHAE* overexpression; sh‐14‐3‐3ε, GA‐D treatment group plus *YWHAE* knockdown; $\bar{x}$ ± SD, mean ± standard deviation. *p < .05, **p < .01.

In Figure 5 legend, there is an error in citing part figure labels. “(c)” should be “(i)”; “(d–f)” should be “(j–l)”; “(g–l)” should be “(c–h)” and the revised caption is provided below.

**FIGURE 5** GA‐D enhanced the defense against oxidative stress on the sera in *D‐gal*‐caused aging mice. (a) The experimental design. (b) The swimming test of mice in the different groups. **(c–h)** Activity of T‐AOC, SOD, MDA, GSH‐Px, AGEs, and RAGE in the sera of mice exposed to different treatments. **(i)** Histopathological organization of the liver, kidney, and heart tissues in different mice groups, analyzed via hematoxylin–eosin (HE) staining. Scale bar: 200 μm. **(j–l)** The histological scores of liver, kidney, and heart tissues in different mouse groups. *n* = 6. AGEs, advanced glycation end products; GSH‐Px, glutathione peroxidase; igGG‐H, in vivo high‐dose GA‐D treatment group; igGG‐L, in vivo low‐dose GA‐D treatment group; igGG‐M, in vivo medium‐dose GA‐D treatment group; MDA, malondialdehyde; MG, model group (*D‐gal*‐caused aging mice); NG, normal group; RAGEs, receptor for advanced glycation end products; SG, solvent group (*D‐gal*‐caused aging mice treated with 0.1% DMSO via intragastric administration); SOD, superoxide dismutase; T‐AOC, total antioxidant capacity; $\bar{x}$ ± SD, mean ± standard deviation. *p < .05, **p < .01.

In addition, the published version of **Supporting information** files has missing figure captions and the revised supporting information along with their captions has been included.

## Supporting information


Supplementary Material
