# Supplementary material for: Ganoderic acid D prevents oxidative stress‐induced senescence by targeting 14‐3‐3ε to activate CaM/CaMKII/NRF2 signaling pathway in mesenchymal stem cells
Source: Aging Cell. 2022 Aug 5;21(9):e13686. doi: 10.1111/acel.13686 (PMC9470892; doi:10.1111/acel.13686)

Supplementary Figure_1.tiff

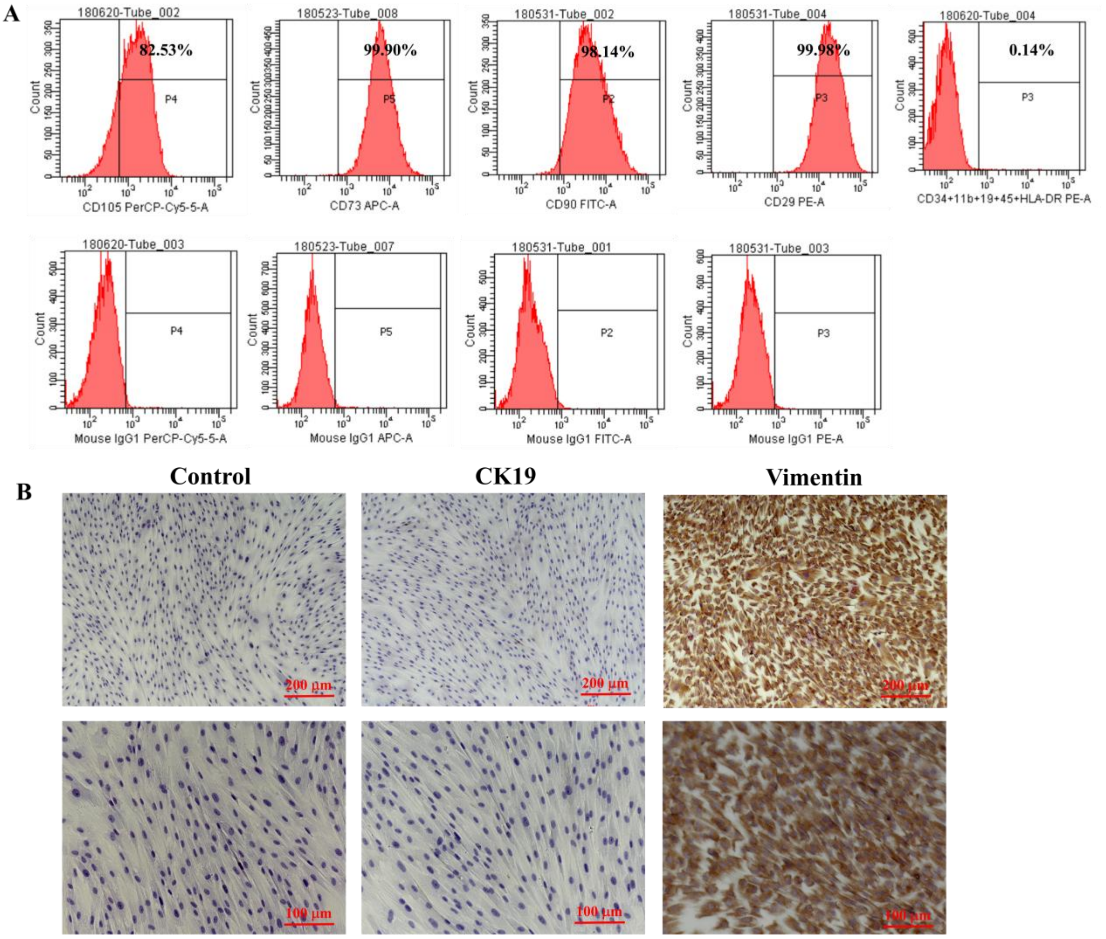

Supplementary Figure_2.tiff

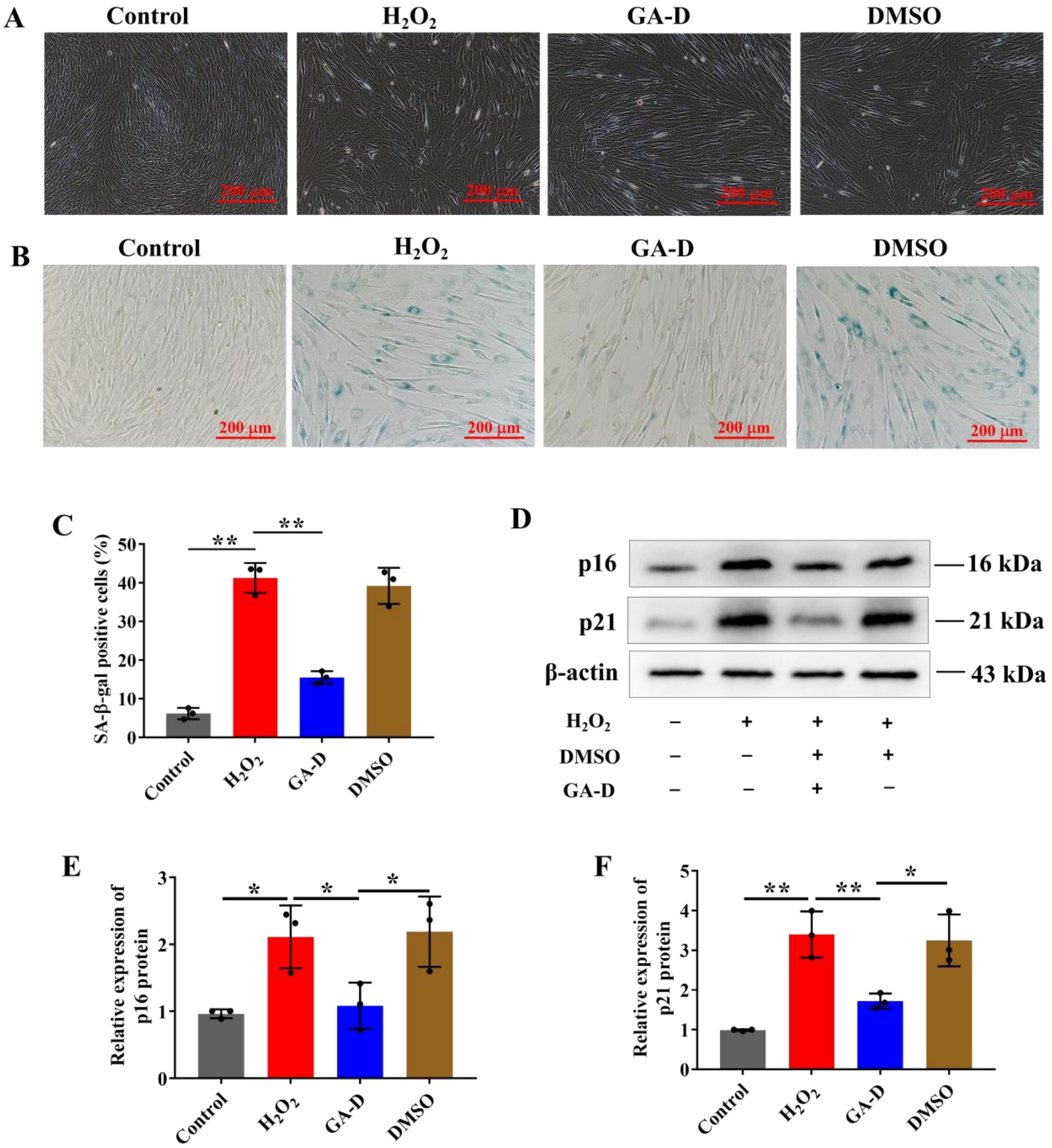

Supplementary Figure_3.tiff

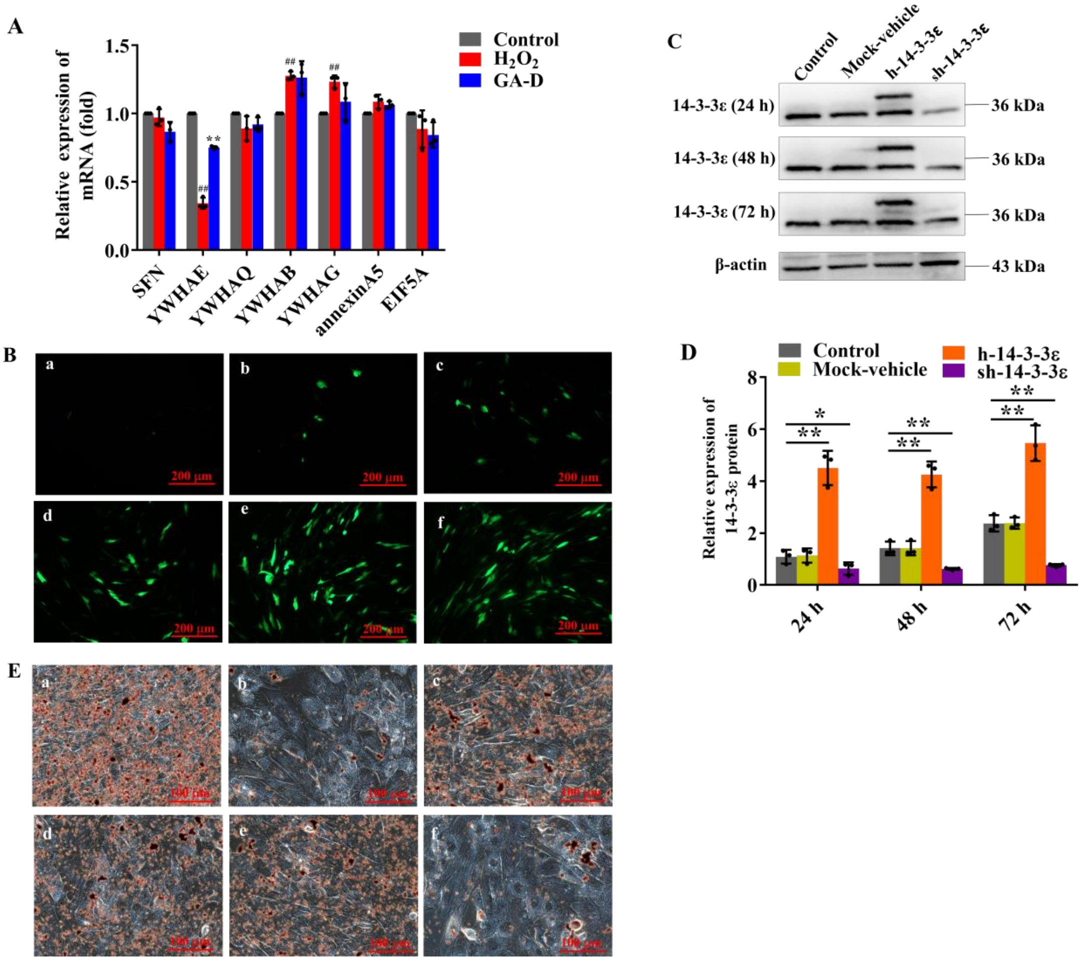

Supplementary Figure_4.tiff

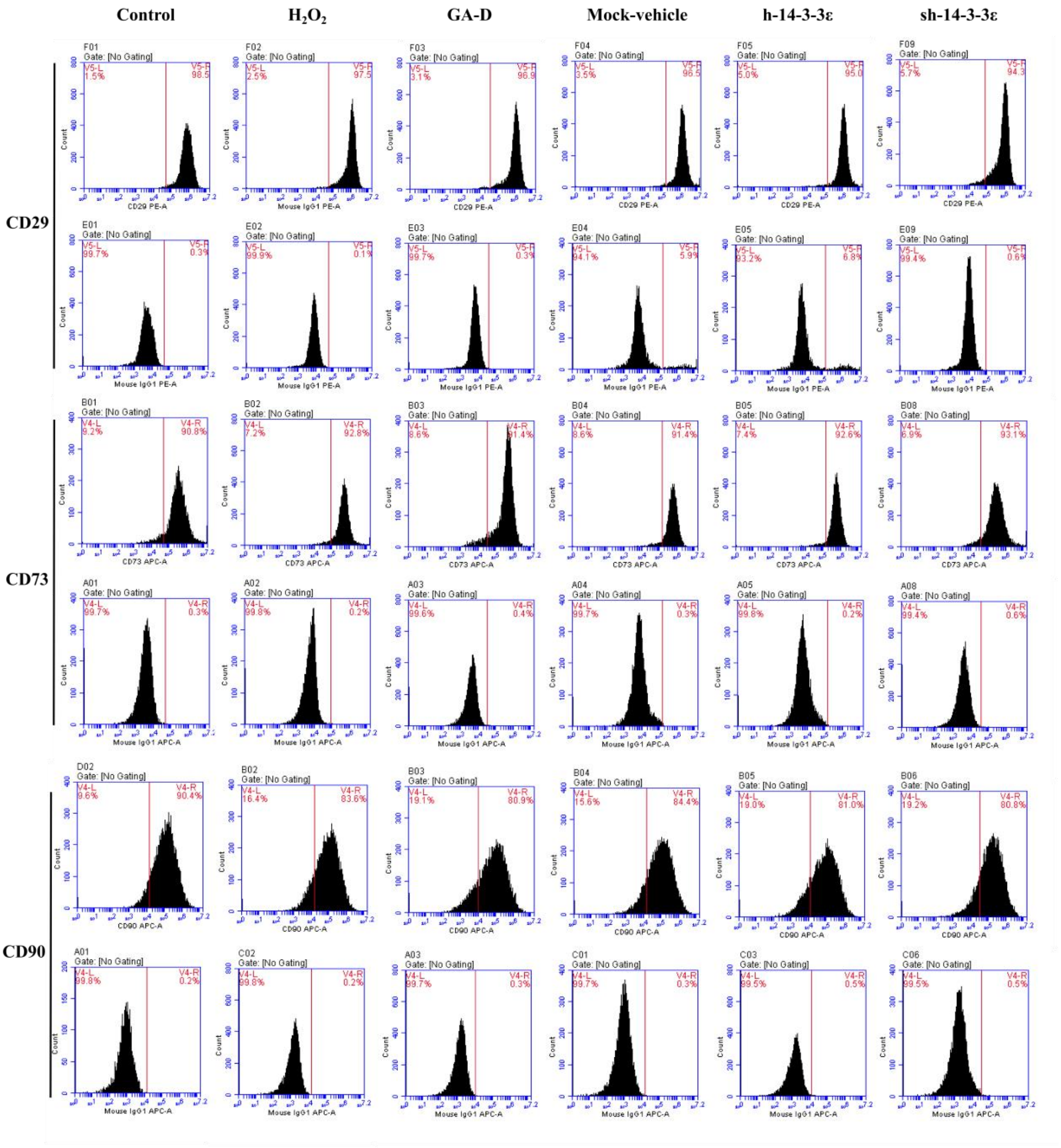

Supplementary Figure_5.tiff

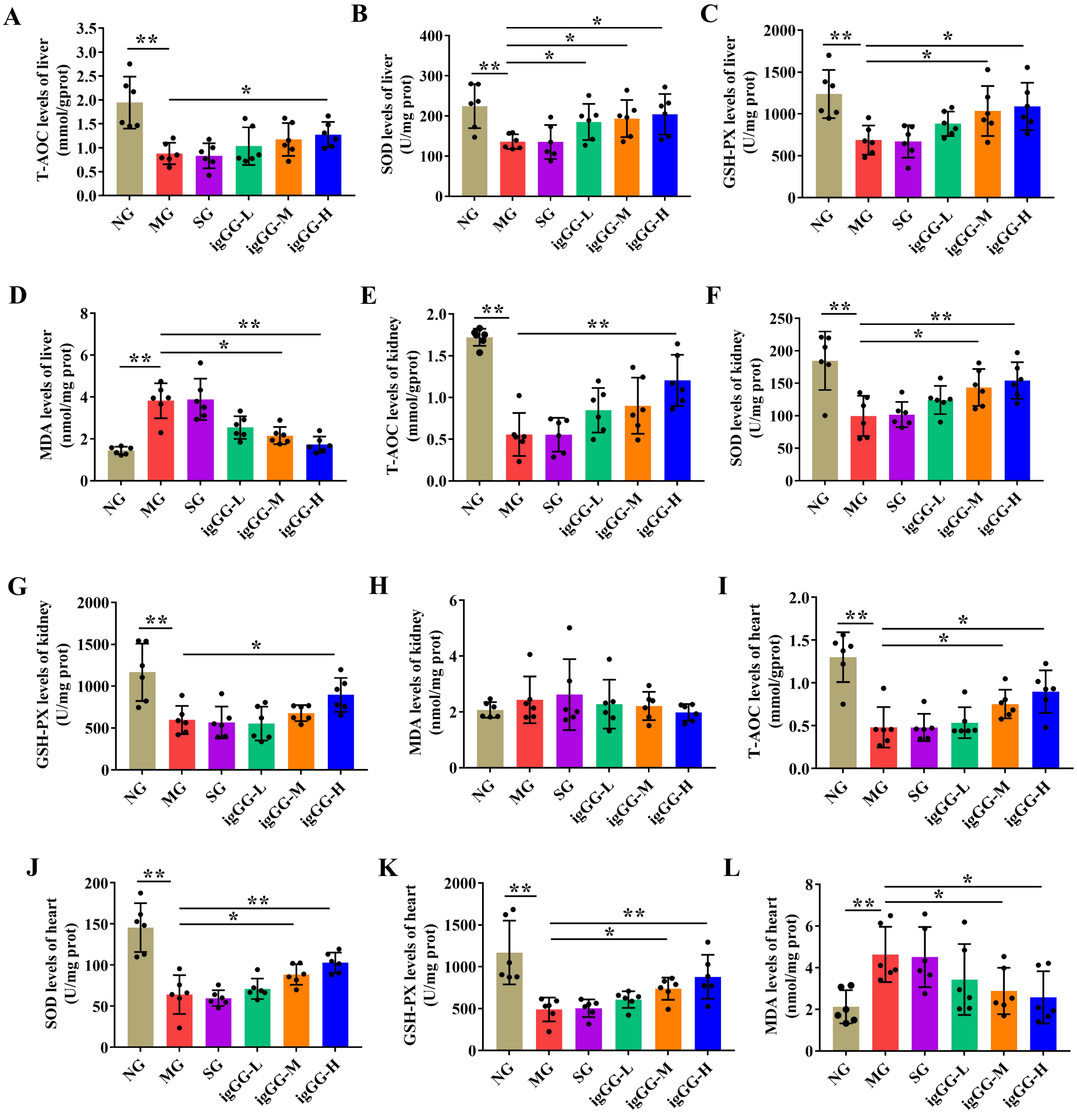

Supplementary Figure_6.tiff

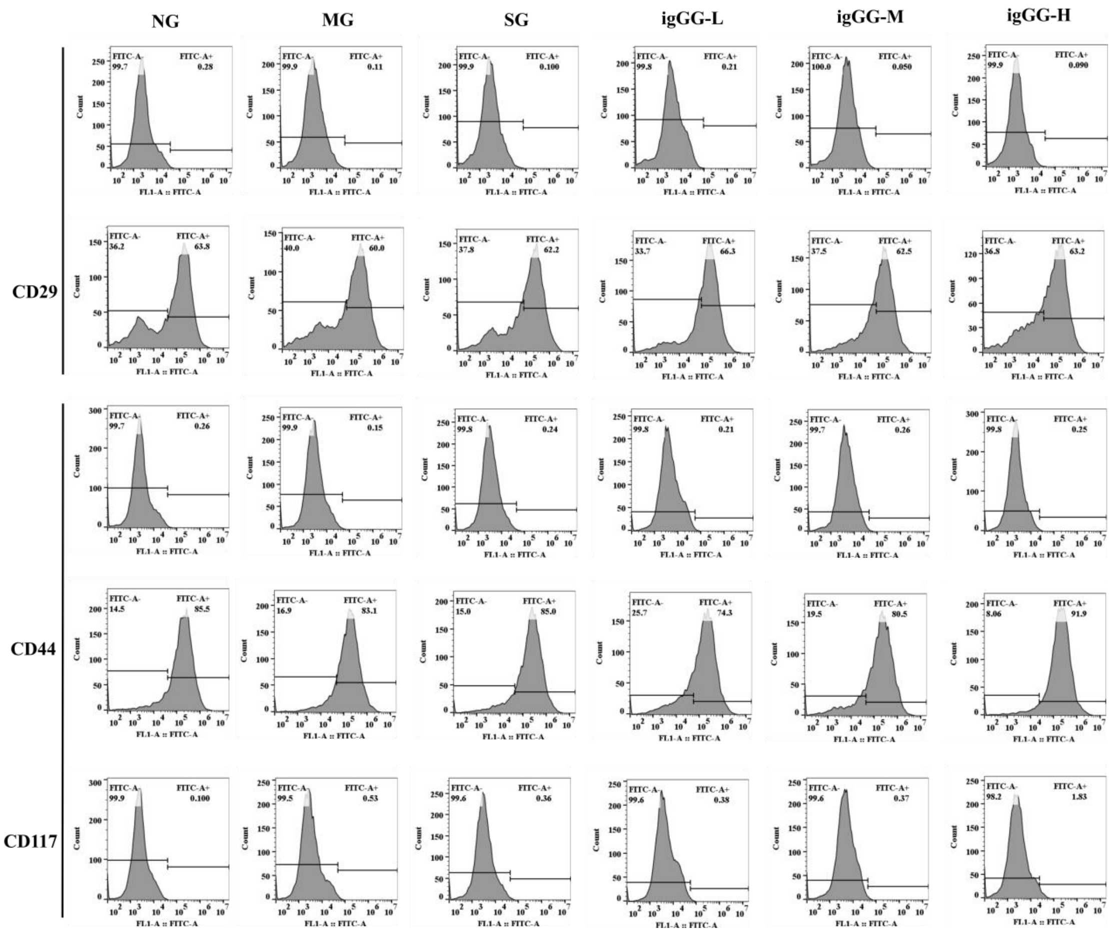

Supplementary Figure_7.tiff

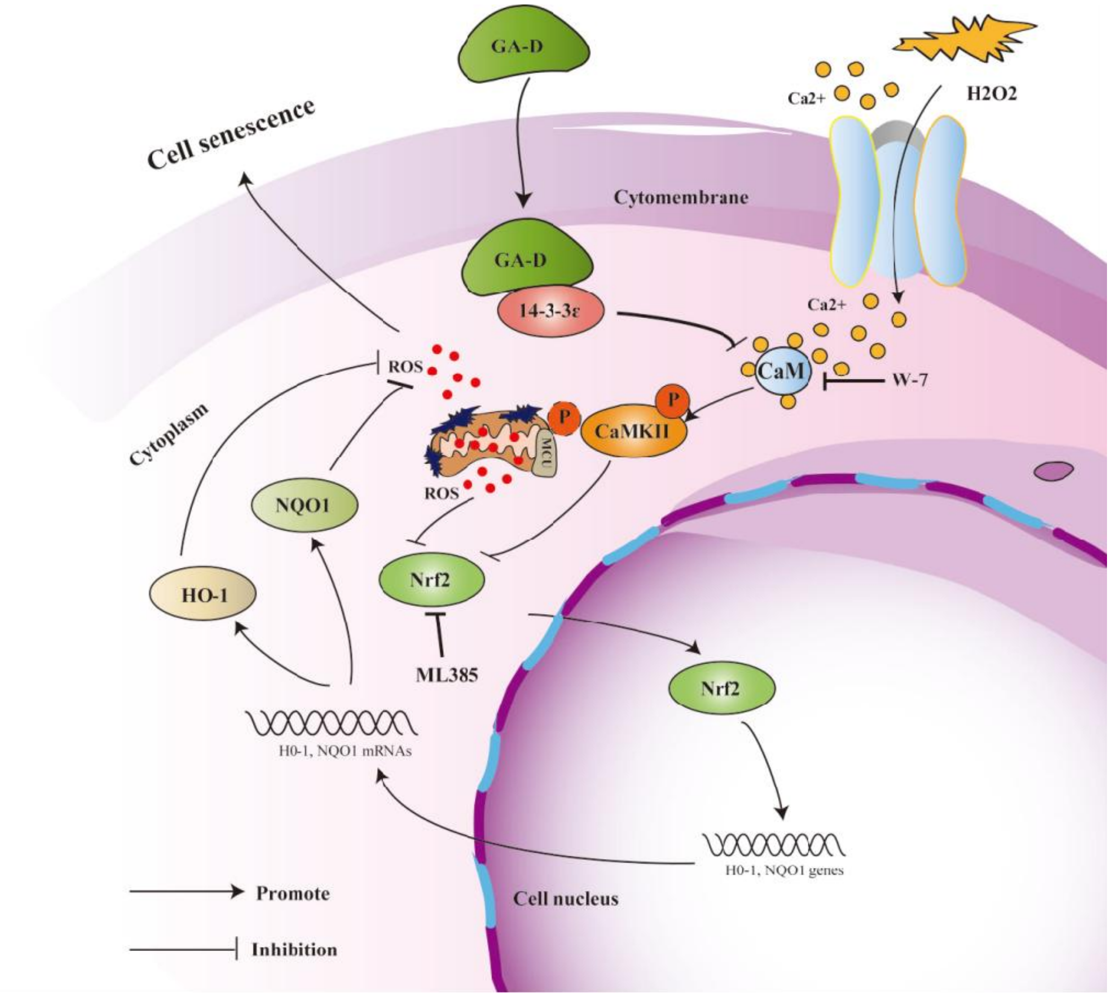

Supplement: Supplementary file 1 — Figure S1 Figure S2 Figure S3 Figure S4 Figure S5 Figure S6 Figure S7 [file ACEL-21-e13686-s001.pdf]
