# Supplementary material for: Ganoderic acid D prevents oxidative stress‐induced senescence by targeting 14‐3‐3ε to activate CaM/CaMKII/NRF2 signaling pathway in mesenchymal stem cells
Source: Aging Cell. 2022 Aug 5;21(9):e13686. doi: 10.1111/acel.13686 (PMC9470892; doi:10.1111/acel.13686)
Supplement: Supplementary file 2 — Table S1 [file ACEL-21-e13686-s002.docx]

**Supplementary Table 1. Primer sequences of target genes**

| Gene | Sequence（5′→3′） | Genbank ID | Length of product (bp) |
| --- | --- | --- | --- |
| *YWHAE*  (14-3-3ε) | For: GCAGGGTGACGATTCCTA  Rev:GGGGTGGTCAGAGATGG | NM_169798.3 | 149 |
| *SFN*  (14-3-3σ) | For: GTTGAGCGCACCTAACCA  Rev: GGAGGGAGGCAAGAGGA | NM_001135699.2 | 105 |
| *YWHAQ*  (14-3-3θ) | For: CTGAAGTTGCGTGTGGTG  Rev: GGATTGGGTGTGTGGGT | NM_006826.4 | 111 |
| *YWHAB*  (14-3-3β/α) | For: TCTGAAGTGGCATCTGGA  Rev: GACGAATTGGGTGTGTAGG | NM_003404.5 | 115 |
| *YWHAG*  (14-3-3γ) | For: ACTGGGGCGTTTCCTACT  Rev: CACACTGCACCACAGACC | NM_012479.4 | 100 |
| *annexin A5* | For: TCCCTGGATTTGATGAGC  Rev:TGAGCATTACTTCGGGATG | NM_001154.4 | 112 |
| *EIF5A* | For: AATGGCTTTGTGGTGCTC  Rev: ATGAGTTGACGGGCAGATA | NM_001143760.1 | 157 |
| *β-actin* | For: TGGCACCCAGCACAATGAA  Rev: CTAAGTCATAGTCCGCCTAGA  AGCA | [NM_001101.3](http://www.ncbi.nlm.nih.gov/entrez/viewer.fcgi?db=nucleotide&id=168480144) | 186 |
