# Supplementary material for: Corrigendum to: Ganoderic acid D prevents oxidative stress‐induced senescence by targeting 14‐3‐3ε to activate CaM/CaMKII/NRF2 signaling pathway in mesenchymal stem cells
Source: Aging Cell. 2022 Oct 19;21(12):e13732. doi: 10.1111/acel.13732 (PMC9741499; doi:10.1111/acel.13732)
Supplement: Supplementary file 1 — Supplementary Material [file ACEL-21-e13732-s001.pdf]

Supplementary Figures and Legends

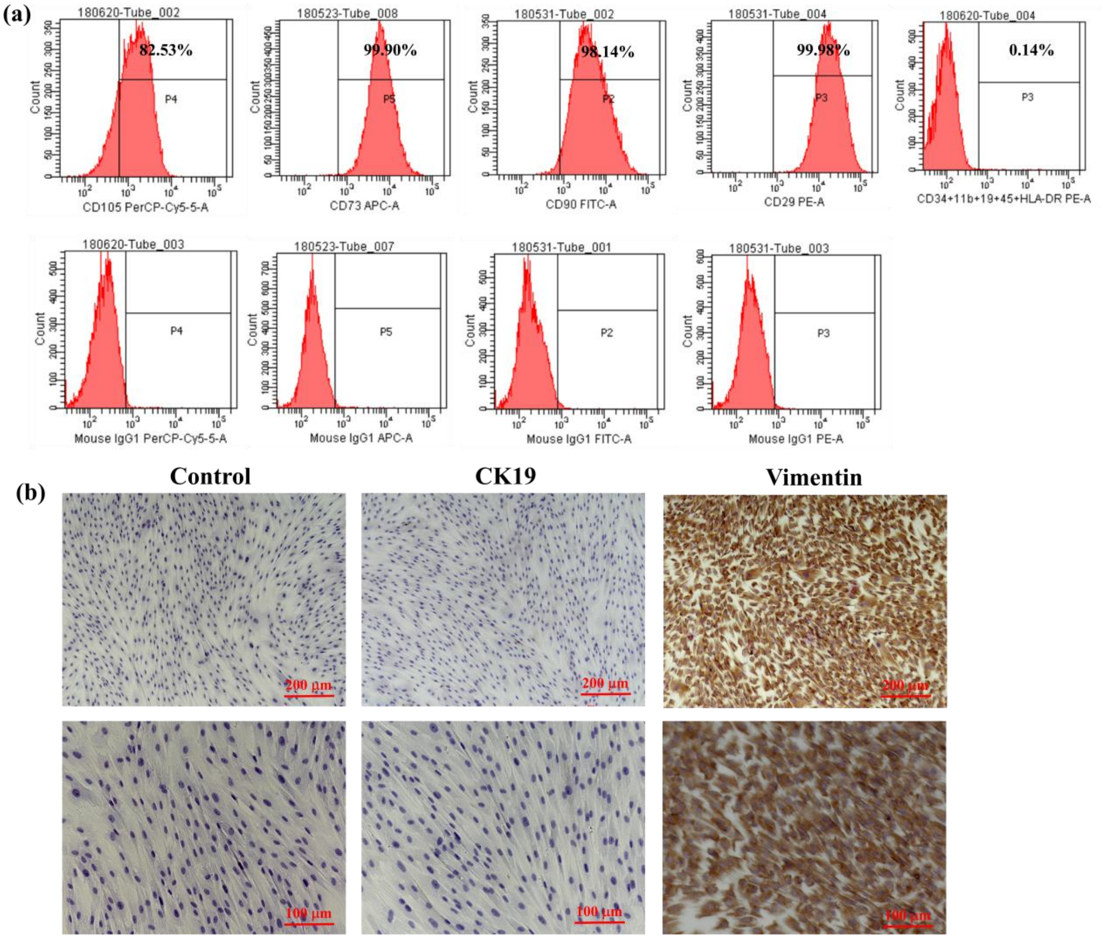

Supplementary Figure 1. Identification of hAMSCs. (a) The expression of molecular markers on the surface of hAMSCs was detected using flow cytometry. (b) Immunocytochemical staining of hAMSCs. Scale bar: 100 μm, 200 μm.

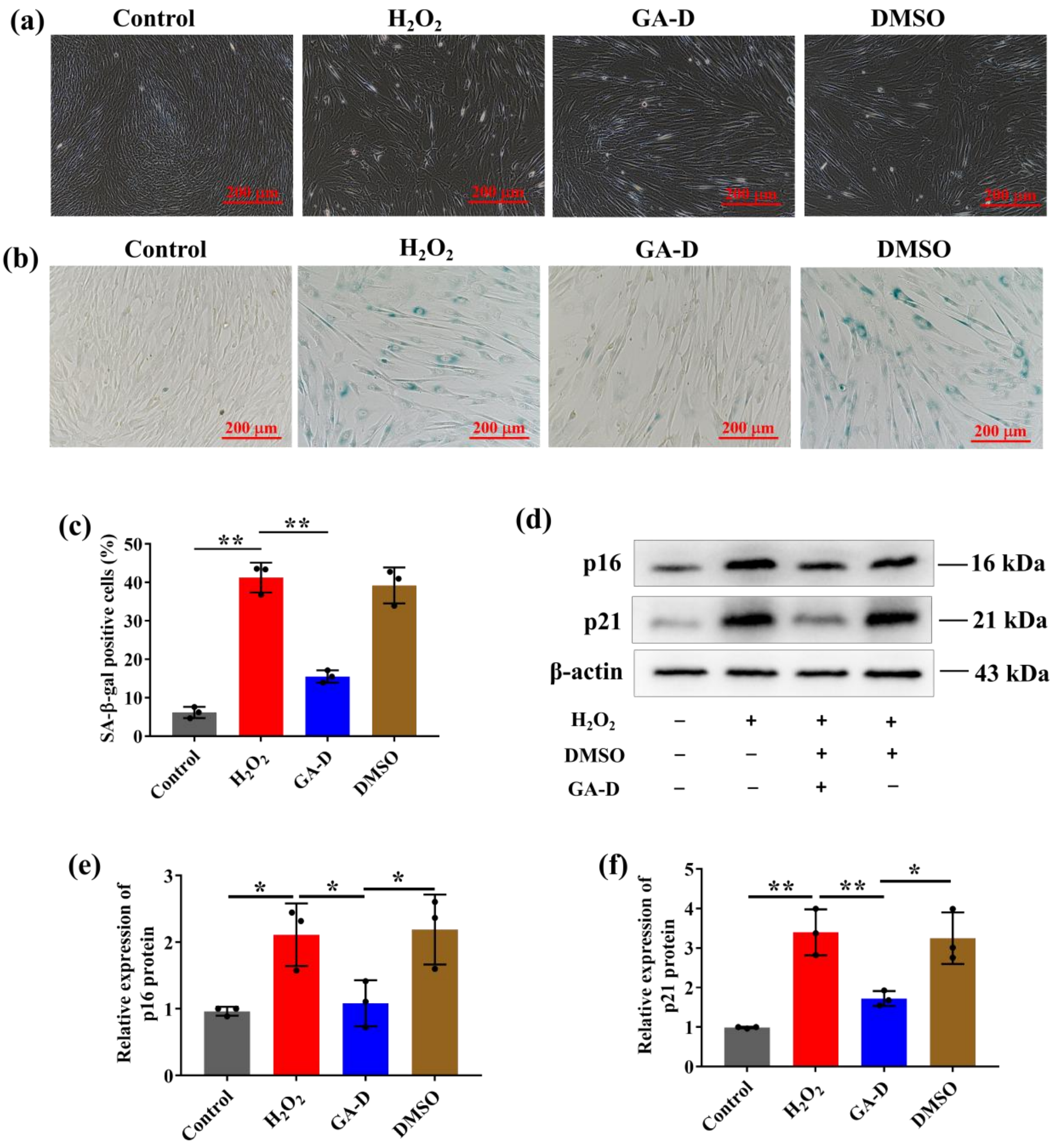

Supplementary Figure 2. The protect effect of GA-D on H_2O_2-induced premature hAMSCs senescence. (a) Effect of GA-D on the morphology of H_2O_2-induced senescent hAMSCs. Scale bar: 200 μm. (b and c) *β*-galactosidase production and percentage of SA-*β*-gal-positive cells after GA-D pretreatment. Scale bar: 200 μm. n = 3. (d) Changes in $p16^{INK4a}$ and p21 expression in hAMSCs. (e and f) Relative expression of $p16^{INK4a}$ and p21. n = 3. Control; control group; H_2O_2, senescent group; GA-D, GA-D treatment group; DMSO, DMSO solvent group; $\bar{x}$ ± sd, mean ± standard deviation. *$P < 0.05$, **$P < 0.01$.

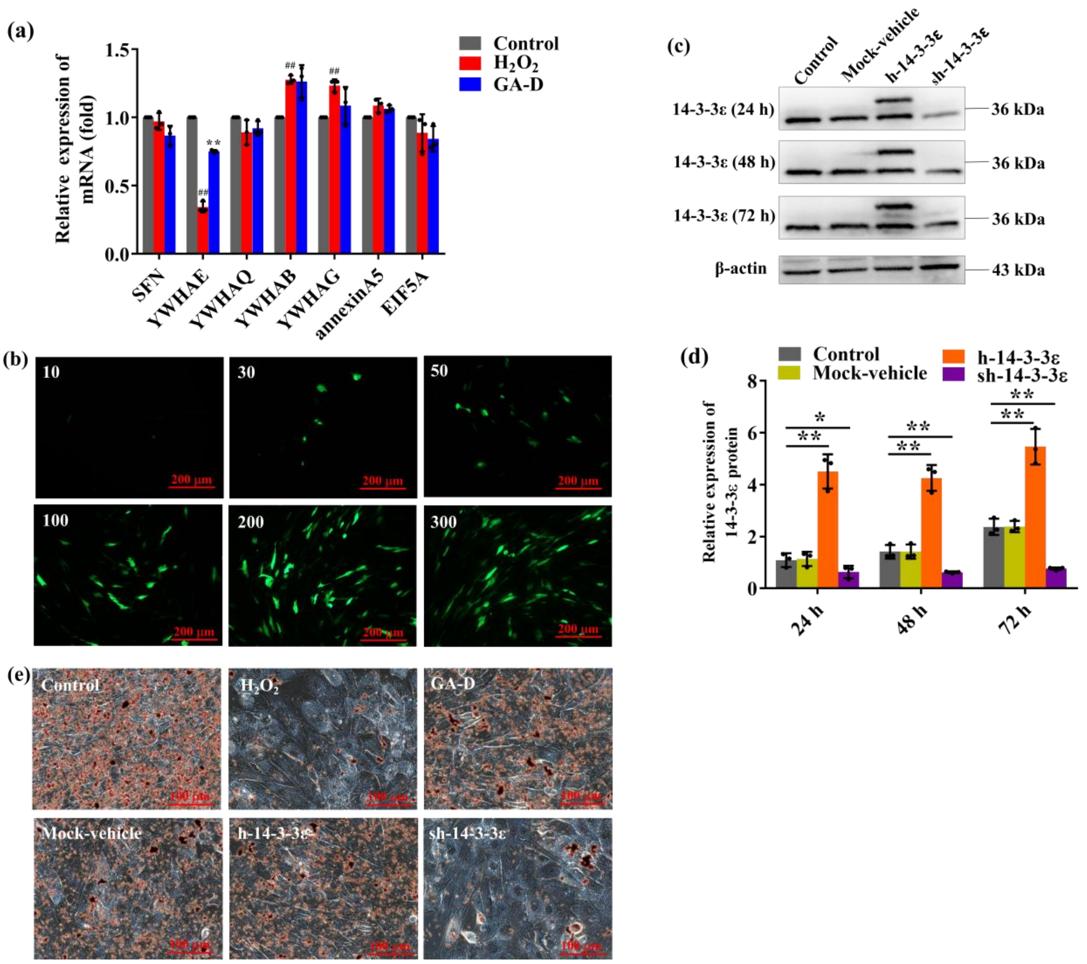

Supplementary Figure 3. The GA-D target *YWHAE* knockdown and overexpression in H_2O_2-induced senescent hAMSCs. (a) Possible target genes associated with GA-D treatment H_2O_2-induced senescent hAMSCs. (b) The multiplicity of infection (MOI) after the viral infection of hAMSCs was set to 10, 30, 50, 100, 200 and 300, respectively. Scale bar: 200 μm. (c and d) the expression of 14-3-3ε protein after overexpression and knockdown of *YWHAE*. Control, control group; Mock-vehicle, empty carrier; h-14-3-3ε, *YWHAE* overexpression; sh-14-3-3ε, *YWHAE* knockdown (e) Oil red O stain staining of hAMSCs. Control, control group; H_2O_2, senescent group; GA-D, GA-D treatment group; Mock-vehicle, GA-D treatment group plus empty carrier; h-14-3-3ε, GA-D treatment group plus *YWHAE* overexpression; sh-14-3-3ε, GA-D treatment group plus *YWHAE* knockdown; n = 3. $\bar{x}$ ± sd, mean ± standard deviation. *$P < 0.05$, **$P < 0.01$.

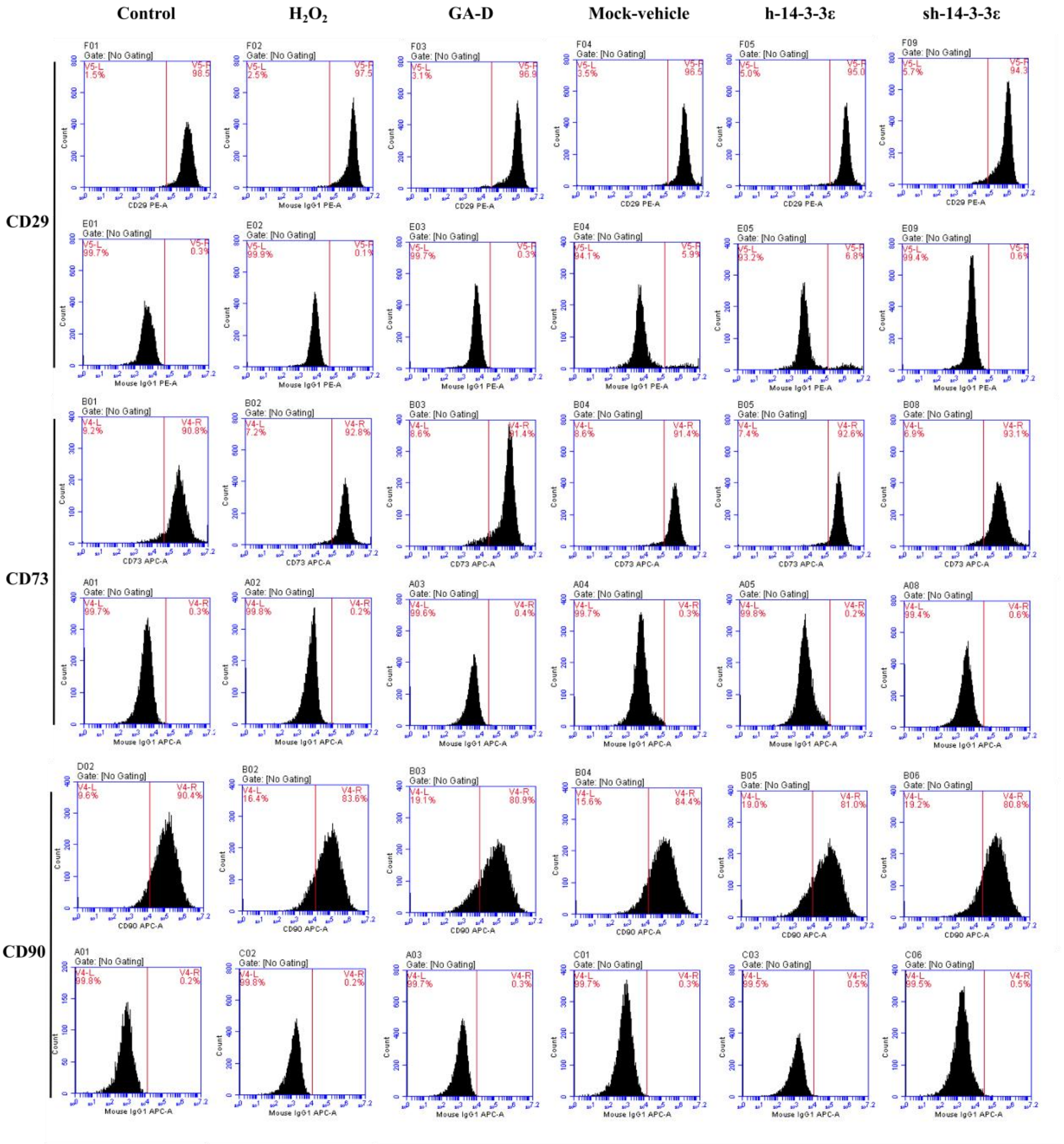

Supplementary Figure 4. The expression of molecular markers on the surface of hAMSCs was detected using flow cytometry after the GA-D target *YWHAE* knockdown and overexpression. Control, control group; H_2O_2, senescent group; GA-D, GA-D treatment group; Mock-vehicle, GA-D treatment group plus empty carrier; h-14-3-3ε, GA-D treatment group plus *YWHAE* overexpression; sh-14-3-3ε, GA-D treatment group plus *YWHAE* knockdown.

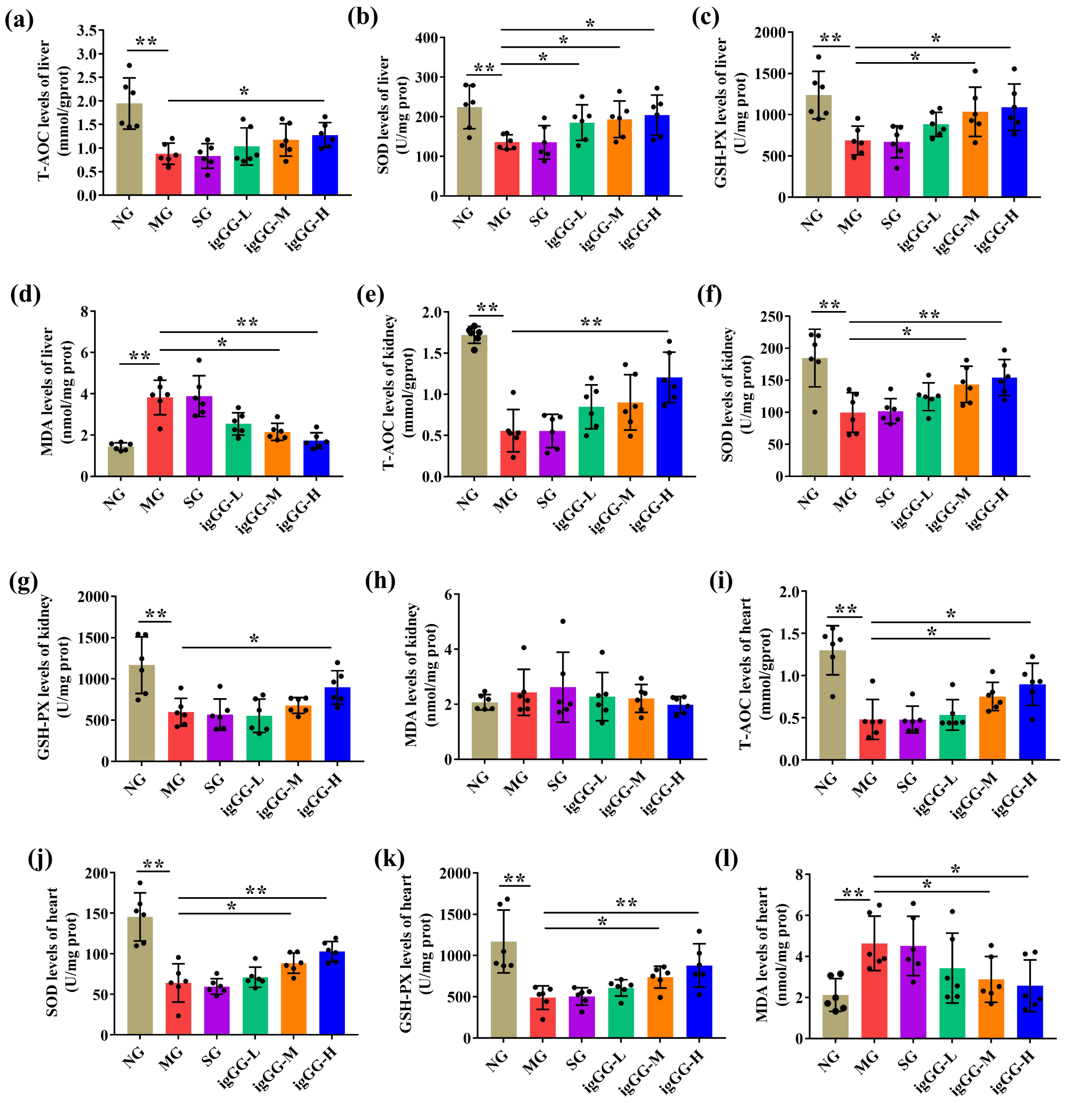

Supplementary Figure 5. GA-D enhanced the defense against oxidative stress in the liver, kidneys, and heart in *D-gal-caused* aging mice. (a–d) Activity of T-AOC, SOD, GSH-Px and MDA in the liver of mice upon different treatments. (e–h) Activity of T-AOC, SOD, GSH-Px, and MDA in the kidneys. (i–l) Activity of T-AOC, SOD, GSH-Px, and MDA in the heart. n = 6. T-AOC, total antioxidant capacity; SOD, superoxide dismutase; GSH-Px, glutathione peroxidase; MDA, malondialdehyde; AGEs, advanced glycation end products; RAGEs, receptor of advanced glycation end-products; NG, normal group; MG, model group (*D-gal*-caused aging mice); SG, solvent group (*D-gal*-caused aging mice treated with 0.1% DMSO via intragastric administration); igGG-L, *in vivo* low-dose GA-D treatment group; igGG-M, *in vivo* medium-dose GA-D treatment group; igGG-H, *in vivo* high-dose GA-D treatment group. $\bar{x}$ ± sd, mean ± standard deviation. **P* < 0.05, ***P* < 0.01.

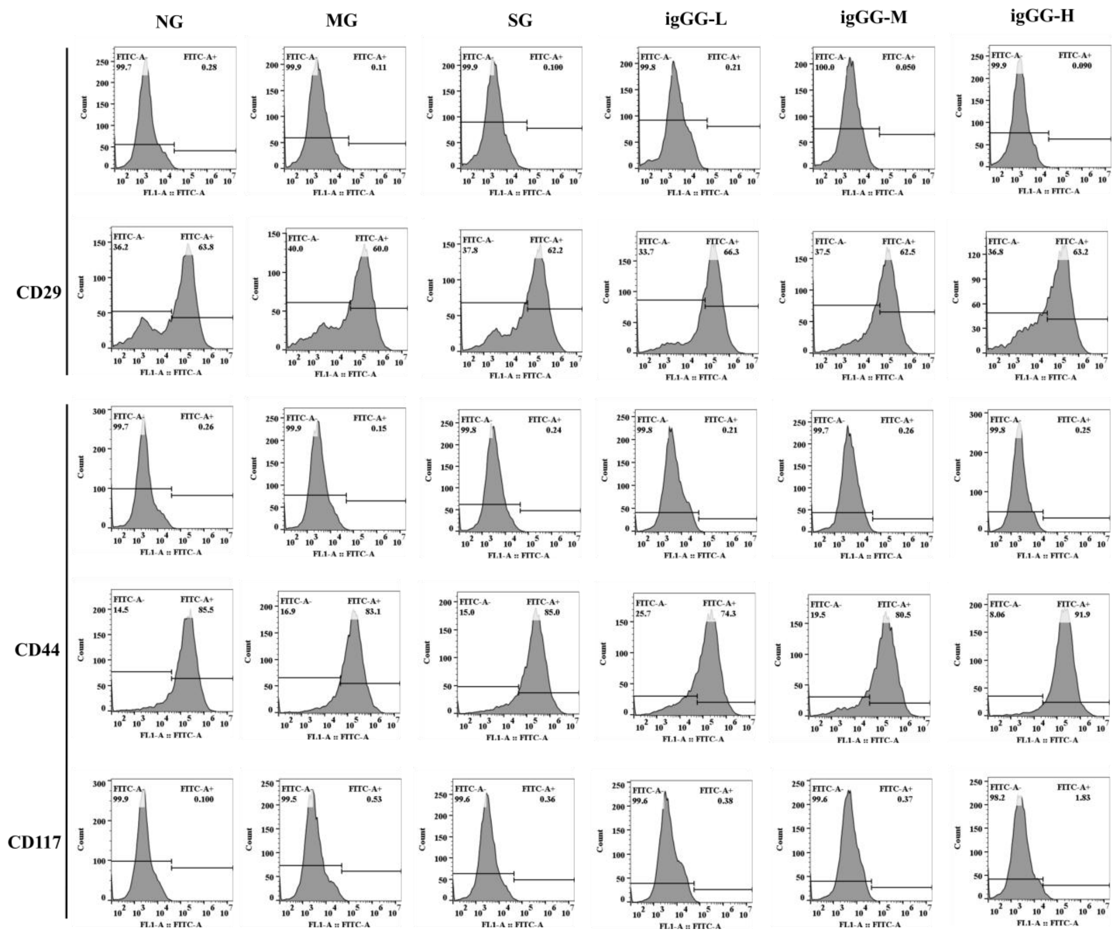

Supplementary Figure 6. The expression of molecular markers on the surface of BMSCs isolated from mice in each groups was detected using flow cytometry. NG, normal group; MG, model group (*D*-gal-caused aging mice); SG, solvent group (D-gal-caused aging mice treated with 0.1% DMSO via intragastric administration); igGG-L, *in vivo* low-dose GA-D treatment group; igGG-M, *in vivo* medium-dose GA-D treatment group; igGG-H, in vivo high-dose GA-D treatment group.

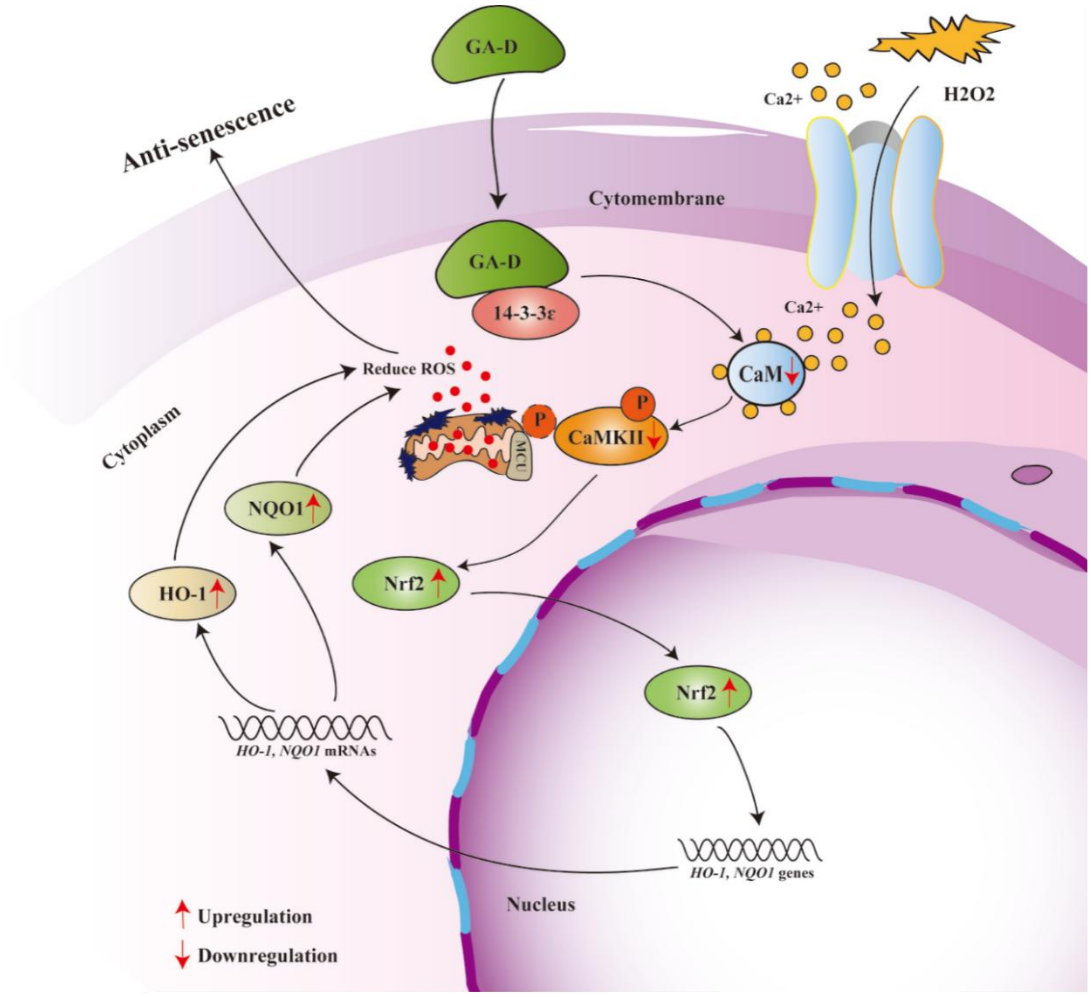

Supplementary Figure 7. A schematic diagram showing the proposed anti-senescence mechanism of GA-D in hAMSCs. Here GA-D downregulates CaM and phosphorylated CaMKII through targeting the 14-3-3ε isoform, which maintains calcium homeostasis and promotes intranuclear transfer of Nrf2, as well as HO-1 and NQO1 expression, which in turn inhibits ROS formation to finally prevent hAMSC senescence. *P*, phosphorylation; 14-3-3ε, 14-3-3 epsilon; CaM, Calmodulin; CaMKII, Ca2+/calmodulin dependent protein kinase II; Nrf2, Nuclear Factor erythroid 2-Related Factor 2; HO-1, hemeoxygenase-1; NQO1, NAD(P)H: quinoneoxidoreductase; ROS, Reactive oxygen species.
